# Quantifying the Influence of Covalent Metal‐Ligand Bonding on Differing Reactivity of Trivalent Uranium and Lanthanide Complexes

**DOI:** 10.1002/anie.202211145

**Published:** 2022-10-12

**Authors:** Taylor V. Fetrow, Joshua Zgrabik, Rina Bhowmick, Francesca D. Eckstrom, George Crull, Bess Vlaisavljevich, Scott R. Daly

**Affiliations:** ^1^ Department of Chemistry The University of Iowa E331 Chemistry Building Iowa City IA 52242 USA; ^2^ Department of Chemistry The University of South Dakota 414 East Clark Street Vermillion South Dakota 57069 USA

**Keywords:** Actinide, Borohydride, Covalency, Density Functional Theory, Lanthanide

## Abstract

Qualitative differences in the reactivity of trivalent lanthanide and actinide complexes have long been attributed to differences in covalent metal‐ligand bonding, but there are few examples where thermodynamic aspects of this relationship have been quantified, especially with U^3+^ and in the absence of competing variables. Here we report a series of dimeric phosphinodiboranate complexes with trivalent *f*‐metals that show how shorter‐than‐expected U−B distances indicative of increased covalency give rise to measurable differences in solution deoligomerization reactivity when compared to isostructural complexes with similarly sized lanthanides. These results, which are in excellent agreement with supporting DFT and QTAIM calculations, afford rare experimental evidence concerning the measured effect of variations in metal‐ligand covalency on the reactivity of trivalent uranium and lanthanide complexes.

## Graphical abstract

Here we report experimental and computational studies that quantify the measured effect of variations in metal‐ligand covalency on the reactivity of phosphinodiboranate complexes with trivalent uranium and similarly sized lanthanides.

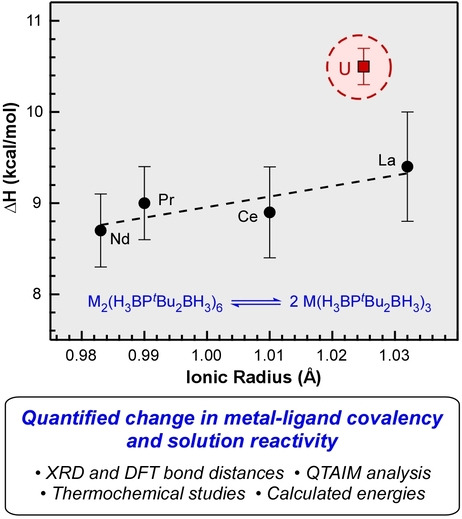

One of the most critical debates in f‐element science continues to center on the role of covalent metal‐ligand bonding on the reactivity of trivalent lanthanide and actinide complexes.^[1]^ As proposed by Seaborg and co‐workers in the 1950′s,^[2]^ suspected differences in covalent metal‐ligand bonding with 4*f* and 5*f* metals are often invoked when attempting to account for reactivity differences observed with trivalent actinides and lanthanides,^[3]^ especially in processes related to separations and ligand binding studies.^[4]^ Prominent examples reported by Jensen, Nash, and co‐workers, for example, ascribed Am^3+^/Ln^3+^ binding enthalpy differences of 2.1–2.8 kcal mol^−1^ to increased metal‐ligand covalency with multidentate N‐donor ligands forming three to six M−N bonds.^[3a, 3b]^ Moreover, Shafer, Yang, and co‐workers attributed the ca. 1 kcal mol^−1^ free energy differences for stepwise binding of picolinate ligands to heavy trivalent actinides to energy degeneracy driven covalency.^[5]^

While the existence of covalency in certain trivalent actinide‐ligand bonds is settled,^[6]^ questions loom as to whether covalency differences with respect to lanthanides are large enough to account for observed reactivity differences, especially for studies conducted in aqueous/organic mixtures.^[1c, 7]^ It has been shown, for example, how competing variables (e.g., hydration, entropic effects) could account for some empirical differences in reactivity.^[8]^ Adding to this challenge, speciation is often poorly defined in aqueous mixtures,^[9]^ making it difficult to quantify thermodynamic values for all the complexes that may be involved. Another challenge is identifying experimental hallmarks of covalency that correlate clearly to differences in reactivity.^[10]^

As illustrated in the examples above, aqueous reactivity comparisons with trivalent actinides are typically limited to transplutonium elements because early actinides like U^3+^ are unstable with respect to oxidation in air and water.^[11]^ Indeed, investigations of covalency‐induced reactivity and structure differentiation between trivalent uranium and lanthanides have come from non‐aqueous studies,^[12]^ especially with metallocene complexes.^[13]^ However, even in these systems, quantifying thermodynamic changes in reactivity as they pertain to differences in metal‐ligand covalency remains a challenge.

In this context, it has been proposed that trivalent lanthanide and uranium complexes containing borohydride ligands such as DMADB (H_3_BNMe_2_BH_3_^−^) can give rise to covalency‐induced differences in reactivity. Pr(H_3_BNMe_2_BH_3_)_3_ and U(H_3_BNMe_2_BH_3_)_3_, for example, are isostructural coordination polymers in the solid state, but Pr(H_3_BNMe_2_BH_3_)_3_ readily depolymerizes and sublimes when heated under vacuum whereas U(H_3_BNMe_2_BH_3_)_3_ does not (Scheme 1a).^[14]^ Subsequent theoretical studies supported that the dramatic differences in the volatility of U(H_3_BNMe_2_BH_3_)_3_ and Ln(H_3_BNMe_2_BH_3_)_3_ complexes can be attributed to increased covalent U−H−B bonding in bridging DMADB ligands that prevents depolymerization required for U(H_3_BNMe_2_BH_3_)_3_ to sublime.^[15]^ However, as is often the case, there are no experimental data to clearly corroborate and quantify the measured influence of covalent metal‐ligand bonding differences on the observed reactivity.

**Scheme 1:**
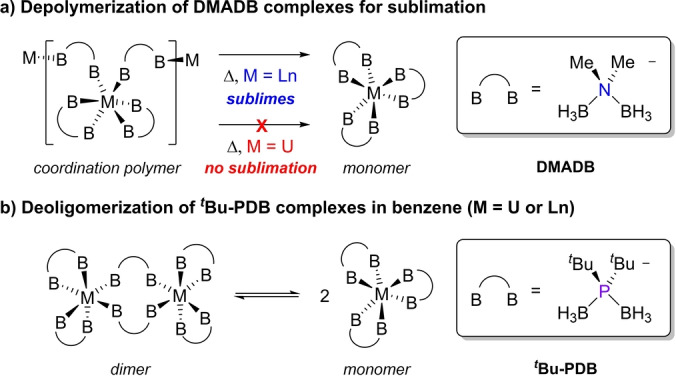
Reactivity comparison of trivalent lanthanide (Ln) and uranium complexes with DMADB and ^*t*^Bu‐PDB.

Phosphorus congeners of aminodiboranates like DMADB called ditertbutylphosphinodiboranates (^*t*^Bu‐PDB) can also be used to prepare homoleptic complexes with trivalent lanthanides and uranium (Scheme 1b),^[16]^ and we show here that their solution reactivity is more amenable to evaluating the influence of metal‐ligand covalency variations. Preliminary studies of M_2_(H_3_BP^*t*^Bu_2_BH_3_)_6_ with M=U, Nd, and Er showed that these complexes exist as dimers in the solid‐state regardless of the metal size but depolymerize to different extents when dissolved in aromatic solvents such as benzene. Our initial studies of M_2_(H_3_BP^*t*^Bu_2_BH_3_)_6_ complexes revealed that they are difficult to prepare using conventional solution‐based salt elimination reactions, which impeded subsequent efforts to investigate their bonding and thermochemical properties thoroughly. Recently, we overcame this obstacle and showed that M_2_(H_3_BP^*t*^Bu_2_BH_3_)_6_ and related phosphinodiboranate complexes can be prepared more reproducibly in higher yields using mechanochemical methods.^[17]^ This has provided reliable access to these complexes for subsequent analysis.

Here, then, we report the mechanochemical synthesis and structures of M_2_(H_3_BP^*t*^Bu_2_BH_3_)_6_ with a complete series of lanthanides (M=La^3+^, Ce^3+^, Pr^3+^, Nd^3+^, Sm^3+^) and U^3+^ that allowed us to quantify the extent to which the identity of the metal ion influences the energy of deoligomerization in solution. The experimental results, which are corroborated by supporting density functional theory (DFT) calculations, quantify small but significant differences between the lanthanides and U^3+^ in their covalent metal‐ligand bonding, correlating to measurable differences in their solution reactivity.

The ^*t*^Bu‐PDB complexes were prepared by grinding three equivalents of K(H_3_BP^*t*^Bu_2_BH_3_) with one equivalent of LnI_3_ (Ln=La–Nd), SmBr_3_, or UI_3_(thf)_4_ using stainless steel balls. The complexes were isolated by extracting the ground solid‐state mixture with Et_2_O or chlorobenzene and subsequently crystallized from pentane mixtures.^[18]^ All six complexes were isolated as single crystals in ≥40 % yield except for La_2_(H_3_BP^*t*^Bu_2_BH_3_)_6_, which gave consistently lower yields (<10 %). It is not clear why the yield was lower with La, but similar results have been reported for the synthesis of La(H_3_BNMe_2_BH_3_)_3_(thf) from LaCl_3_, as compared to the same reaction with other lanthanide chlorides.^[12]^

Single‐crystal XRD studies of the new ^*t*^Bu‐PDB complexes revealed that they exist as dimers in the solid state like U_2_(H_3_BP^*t*^Bu_2_BH_3_)_6_ and Nd_2_(H_3_BP^*t*^Bu_2_BH_3_)_6_.^[19]^ As shown in Figure 1, each trivalent metal ion is bound to two chelating and two bridging ^*t*^Bu‐PDB ligands that hold the dimeric structure together. The M−B distances for the chelating ^*t*^Bu‐PDB ligands are on average ca. 0.2 Å longer than those for the bridging ligands, and they suggest that the denticity of the associated BH_3_ groups are κ^2^ and κ^3^, respectively.

**Figure 1:**
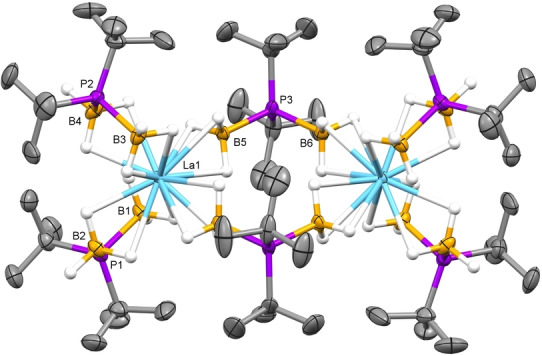
Representative molecular structure of M_2_(H_3_BP^*t*^Bu_2_BH_3_)_6_ (with M=La) obtained from single‐crystal XRD studies. Co‐crystallized pentane and hydrogen atoms attached to carbon were omitted from the figure.

To evaluate the possibility of covalent bonding in M_2_(H_3_BP^*t*^Bu_2_BH_3_)_6_ complexes, we plotted the M−B bond lengths as a function of the ionic radii of the trivalent metal.^[20]^ As described by Raymond and Eigenbrot,^[21]^ f‐metal complexes with metal‐ligand bond lengths that deviate significantly from trends based solely on changes ionic radii are considered to have significant covalent character. As shown in Figure 2, the average M−B distances of the chelating PDB ligands show no significant deviation; the distances are strongly correlated to ionic radii for all the metals including U^3+^ with R^2^=0.98. In contrast, the plot of the average bridging M−B distances vs. ionic radii shows that the bridging U−B distances are ca. 0.04 Å shorter than their expected position based on the line fit to the Ln−B distances (R^2^=0.98). The shorter than expected bridging U−B distances suggest that the U−H−B bonds are more covalent than the Ln−H−B bonds. Consistent with this analysis, we have shown that bond length differences as small as 0.04 Å can give rise to measurable differences in metal‐ligand covalency using ligand K‐edge XAS.^[22]^ Moreover, the 0.04 Å difference is comparable to the 0.036 Å difference reported recently by Goodwin et al. for more covalent U−Se bonds compared to La−Se in M[N(Se=PPh_2_)_2_]_3_ complexes.^[23]^

**Figure 2:**
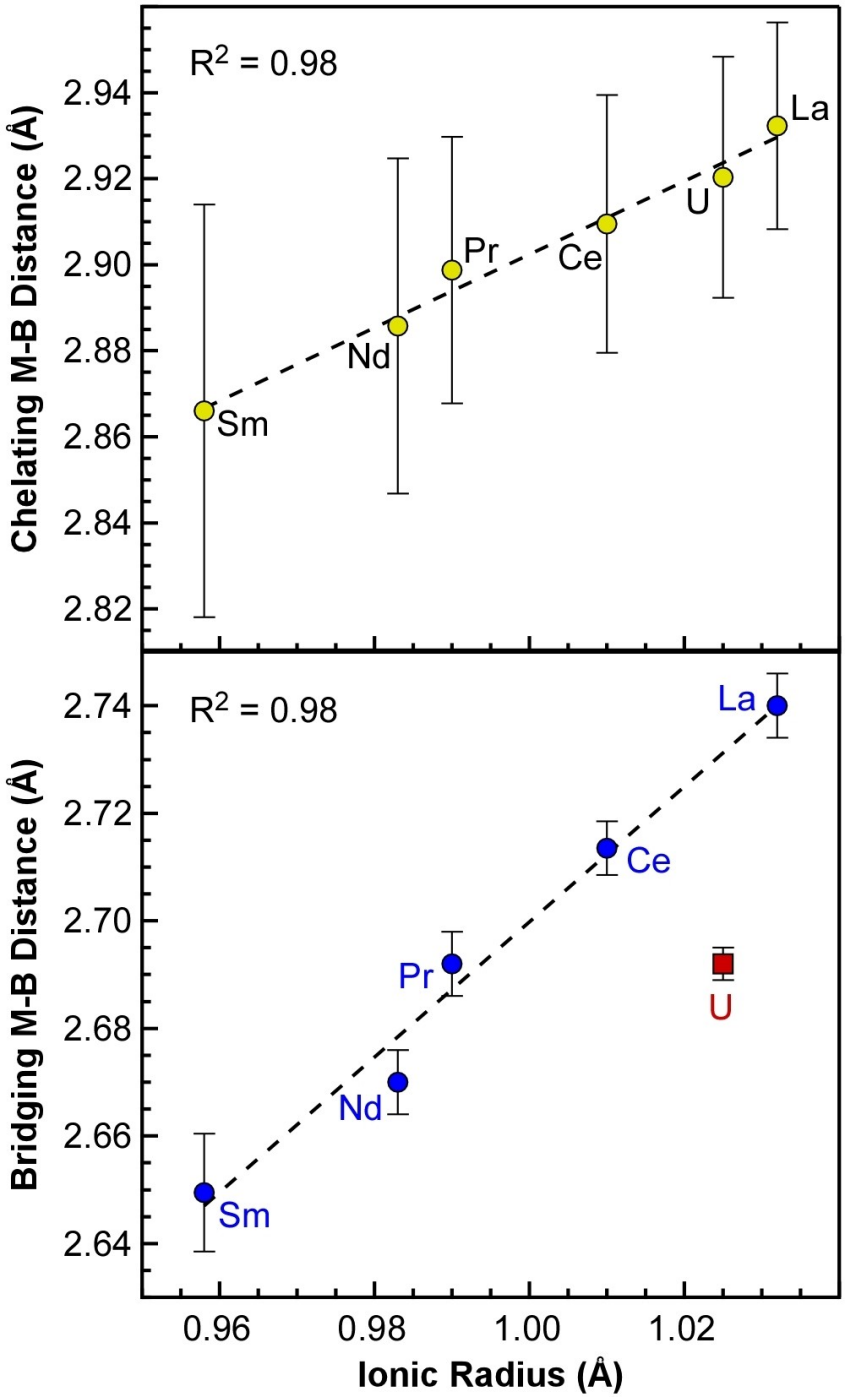
Plot of average chelating (top) and bridging (bottom) M−B distances from single‐crystal XRD data for ^*t*^Bu‐PDB complexes vs. metal ionic radii.^[20]^

Given that the bridging M−H−B bonds are the bonds that must be broken for the M_2_(H_3_BP^*t*^Bu_2_BH_3_)_6_ dimers to convert to monomers, we suspected that the thermochemical values associated with this process should reflect the enhanced covalency in the U−H−B bonds. Variable‐temperature ^1^H NMR studies were therefore carried out on triplicate samples of the complexes with the largest lanthanides (La−Nd) and uranium in C_6_D_6_ to quantify changes in the monomer/dimer concentrations as a function of temperature (Figure 3). The concentrations were used to quantify K_eq_ and values of ΔH, ΔS, and ΔG provided in Table 1 using Van't Hoff plots.

**Figure 3:**
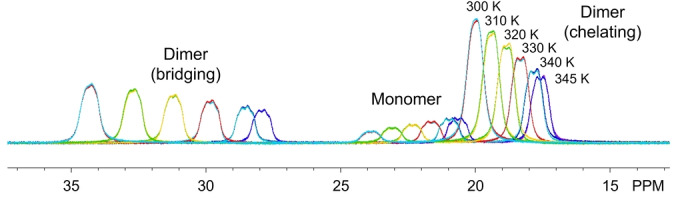
Representative overlay of variable‐temperature ^1^H NMR spectra showing equilibrium mixture of Ce_2_(H_3_BP^*t*^Bu_2_BH_3_)_6_ (dimer) and Ce(H_3_BP^*t*^Bu_2_BH_3_)_3_ (monomer) BH_3_ resonances in C_6_D_6_. Data were collected from 300 K to 345 K and back down to 300 K to demonstrate reversibility.

**Table 1:** Experimental and calculated (TPSS−D3) thermochemical values for the dimer/monomer equilibrium observed with trivalent uranium and lanthanide ^*t*^Bu‐PDB complexes.

M	ΔH [kcal mol^−1^]		ΔS [kcal mol^−1^ K^−1^]		ΔG [kcal mol^−1^]	
	Expt.	DFT	Expt.	DFT	Expt.	DFT
U	10.5 ±0.2	20.6	0.017 ±0.001	0.054	5.3 ±0.2	6.3
La	9.4 ±0.6	18.6	0.016 ±0.002	0.053	4.6 ±0.6	4.8
Ce	8.9 ±0.5	18.0	0.014 ±0.002	0.053	4.7 ±0.5	4.1
Pr	9.0 ±0.4	18.6	0.015 ±0.001	0.055	4.4 ±0.4	4.2
Nd	8.7 ±0.4	17.4	0.016 ±0.001	0.053	4.0 ±0.4	3.6

Plotting the ΔH values obtained from the Van't Hoff plots against the ionic radii of the metals reveals a clear distinction between the U and the lanthanide ^*t*^Bu‐PDB complexes (Figure 4). The ΔH values increase slightly as the size of the lanthanide increases from 8.7±0.4 kcal mol^−1^ with Nd (0.983 Å) to 9.4±0.6 kcal mol^−1^ with La (1.032 Å).^[20]^ The ΔH value for the U complex at 10.5±0.2 kcal mol^−1^ shows a significant departure from the predicted value of 9.2 kcal mol^−1^ based on its ionic radii (1.025 Å) and the linear regression of the lanthanide data points. The increased enthalpy for U indicates that it takes more energy to break up the dimer compared to the lanthanide complexes, which is consistent with the increased metal‐ligand covalency in the bridging ligands.

**Figure 4:**
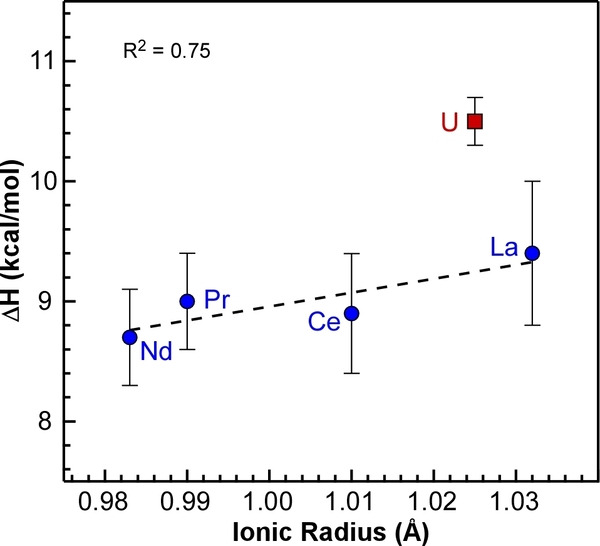
Plot of experimental ΔH from Van't Hoff plots vs. ionic radii of the trivalent metals. Error bars represent standard deviations obtained from triplicate VT NMR measurements. The linear correlation (dotted line) does not include the data point for U_2_(H_3_BP^*t*^Bu_2_BH_3_)_6_.

As shown in Table 1, the experimental ΔS values are all positive and within error, as expected for the increased entropy when breaking the dimers into two monomers. When combined with the ΔH values and temperature, the resulting ΔG is most positive for U at 5.3±0.2 kcal mol^−1^. The difference in ΔG is larger for M=U compared to M=Ce (ΔΔG_U/Ce_=0.6±0.5 kcal mol^−1^) and M=La (ΔΔG_U/La_=0.7±0.6 kcal mol^−1^), but the values are just outside the experimental uncertainties.

We can compare these results to one of the few reports that has quantified the relationship between metal‐ligand bond length and binding enthalpy in trivalent uranium and lanthanide complexes. Ephritikhine, Berthet, and co‐workers reported NMR experiments with U(C_5_H_4_R)_3_ and Ce(C_5_H_4_R)_3_ (where R=^*t*^Bu or SiMe_3_) and various azines (like pyridine) that revealed more exothermic binding to U^3+^ than Ce^3+^ that corresponded to shorter U−N distances compared to Ce−N.^[13c]^ The data varied slightly depending on the ligands used (Table 2), but averaging across the data sets suggest that a 0.029 Å shorter‐than‐expected U−N distance corresponds to a 1.8 kcal mol^−1^ (8 kJ mol^−1^) increase in binding enthalpy. Despite the significant difference in ligands, these values are remarkably consistent with the values reported here for the M_2_(H_3_BP^*t*^Bu_2_BH_3_)_6_ complexes with M=U and Ce; the enthalpy difference of 1.6 kcal mol^−1^ (6.7 kJ mol^−1^) corresponds to a M−B difference of 0.022 Å.

**Table 2:** Comparison of M(C_5_H_4_R)_3_+L→M(C_5_H_4_R)_3_L enthalpy differences for M=U and Ce (ΔΔH_U/Ce_) and differences in M−L bond length in structurally characterized M(C_5_H_4_R)_3_L complexes.^[13c, 24]^

R	L	ΔΔH_U/Ce_ [kJ mol^−1^]	ΔΔH_U/Ce_ [kcal mol^−1^]	ΔM‐L_U/Ce_ [Å]
^*t*^Bu	pyridine	5	1.2	0.029
SiMe_3_	pyridine	10	2.4	0.021
^*t*^Bu	picoline	7	1.7	0.008
SiMe_3_	3,5‐dimethylpyrazine	11	2.6	0.033
SiMe_3_	lutidine	5	1.2	0.054
average		8	1.8	0.029
^*t*^Bu‐PDB (this work)		6.7	1.6	0.022

For comparison to the experimental results, DFT calculations^[25]^ were performed in the Turbomole package using the TPSS‐D3 functional in combination with a triple‐ζ quality basis set on the dimers and monomers. Computations with other functionals were performed, and TPSS−D3 was selected due to its good agreement with experimental free energies. No diffraction data is available for the monomeric species; therefore, conformer sampling of the La species was performed.^[26]^ The conductor‐like screening model (COSMO) was employed to account for solvation using the dielectric constant of benzene.^[27]^ Free energies are computed under standard state conditions assuming all species are present in 1 M concentrations (see Supporting Information for computational details). The geometries are in good agreement with experiment and predict significantly shorter bond lengths for bridging U−B bonds (2.617 Å) than Ln−B bonds (avg. 2.726 Å). The calculated thermochemical values for the reaction M_2_(H_3_BP^*t*^Bu_2_BH_3_)_6_→2 M(H_3_BP^*t*^Bu_2_BH_3_)_3_ were also in excellent agreement with experimental values (Table 1). The highest positive Gibb's free energy (+6.3 kcal mol^−1^) is for the U complex corroborating that deoligomerization requires more energy here than for the lanthanides.

To further probe the differences in bonding in the M−B interactions, QTAIM analysis was performed for the U and Ln species. Computed values for the electron density (ρ), Laplacian electron density (∇^2^(ρ)), energy density (E(r)), the ratio of potential energy and Lagrangian kinetic energy (|V(r)|/G(r)), and the so‐called bond degree (BD), which is the ratio E(r)/ρ, at the bond critical points are provided in the Supporting Information.

The positive Laplacian and the negative energy density indicate that these types of bonds have partial covalent character because they are a direct quantification of the charge accumulation in the bonding region. First, we note that charge accumulation is observed for all complexes with average values of ρ ranging from 0.021 to 0.044 a.u. Values of ρ greater than 0.2 a.u. typically are considered to have pronounced covalent character and these complexes are certainly a predominantly ionic interact.^[28]^ However, the electron density is notably larger for uranium, and it is this difference that is apparent in Figure 5. Moreover, a value of |V(r)|/G(r) greater than one suggests partial covalent character consistent with the highly polarize interaction present in these complexes. In particular, the bridging ligands in the dimers reveal a ratio of 1.22 for U compared to 1.09 for La, supporting that the U−B interaction possesses more covalent character (Table S17). This difference also appears in the bond degree where a negative value indicates covalency; the U−B value is more negative than those for the lanthanides (Table S17). Finally, the so‐called delocalization index (δ) is computed and supports an increased covalency in the U−B bonds (Table S23). Although δ is larger for U−B interactions in both the bridging and chelating ligands than the corresponding lanthanide interactions, the difference is most apparent in the bridging ligands (Figure 5). The fact that the bridging ligands have higher δ values than the chelating ligands overall is consistent with the hypothesis that the strength of these interactions is most important for understanding the monomer/dimer equilibrium.

**Figure 5:**
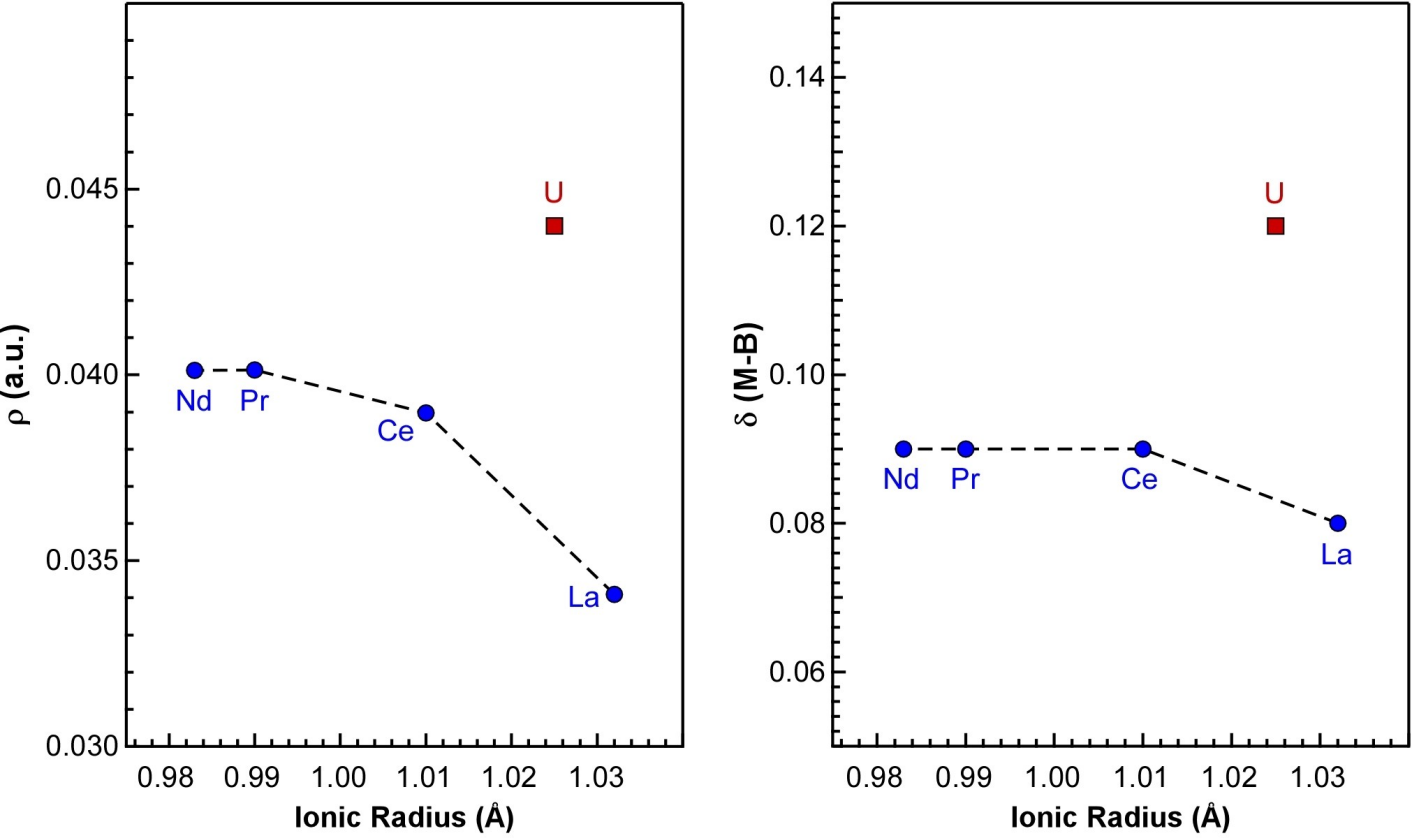
Variation of the calculated electron density (ρ; left) and delocalization index (δ; right) in the bridging ^*t*^Bu‐PDB ligands of the dimers plotted as a function of ionic radii.^[20]^

In summary, we have described a series of trivalent f‐metal phosphinodiboranate complexes that allowed the relationship between covalent metal‐ligand bonding and its associated influence on solution reactivity to be quantified. The shorter‐than‐expected bridging U−B distances in U_2_(H_3_BP^*t*^Bu_2_BH_3_)_6_ correlate to small, but significant increases in the enthalpy and Gibbs free energy required to transform the dimer into monomeric units, as compared to identical complexes with larger and smaller lanthanide ions. Specifically, a 0.04 Å decrease in U−B distance corresponds to a 1.3 kcal mol^−1^ (5.4 kJ mol^−1^) increase in deoligomerization enthalpy based on linear regressions and comparison of ionic radii.

Because the solution studies with M_2_(H_3_BP^*t*^Bu_2_BH_3_)_6_ complexes were conducted on pure complexes in a single non‐polar solvent, the results suggest that differences in deoligomerization enthalpy can be ascribed solely to differences in metal‐ligand covalency. This finding is in excellent agreement with our supporting calculations and appears to provide unambiguous evidence of the quantified influence that metal‐ligand covalency can have on the differing reactivity of trivalent uranium and lanthanides.

## Conflict of interest

The authors declare no conflict of interest.

## Supporting information

As a service to our authors and readers, this journal provides supporting information supplied by the authors. Such materials are peer reviewed and may be re‐organized for online delivery, but are not copy‐edited or typeset. Technical support issues arising from supporting information (other than missing files) should be addressed to the authors.

Supporting Information

Supporting Information

Supporting Information

Supporting Information

Supporting Information

Supporting Information

Supporting Information

Supporting Information

Supporting Information

Supporting Information

Supporting Information

Supporting Information

Supporting Information

Supporting Information

Supporting Information

Supporting Information

Supporting Information

Supporting Information

Supporting Information

Supporting Information

Supporting Information

## Data Availability

The data that support the findings of this study are available in the supplementary material of this article.
