# Supplementary material for: Quantifying the Influence of Covalent Metal‐Ligand Bonding on Differing Reactivity of Trivalent Uranium and Lanthanide Complexes
Source: Angew Chem Int Ed Engl. 2022 Oct 12;61(45):e202211145. doi: 10.1002/anie.202211145 (PMC9828012; doi:10.1002/anie.202211145)
Supplement: Supplementary file 9 — Supporting Information [file ANIE-61-0-s021.pdf]

Angewandte Chemie
Eine Zeitschrift der Gesellschaft Deutscher Chemiker

Supporting Information

Quantifying the Influence of Covalent Metal-Ligand Bonding on Differing Reactivity of Trivalent Uranium and Lanthanide Complexes

T. V. Fetrow, J. Zgrabik, R. Bhowmick, F. D. Eckstrom, G. Crull, B. Vlaisavljevich, S. R. Daly**

SUPPORTING INFORMATION

Table of Contents

1. Synthesis and Characterization 2
2. Thermodynamic Studies 7
3. Crystallographic Studies 16
4. Computational Studies 20
5. NMR Spectra 36
6. IR Spectra 47
7. Supporting Information References 50

1. Synthesis and Characterization

General considerations. All reactions were carried out under an atmosphere of N_2 or Ar using glovebox or standard Schlenk techniques. All glassware was heated at 150 °C for at least two hours and allowed to cool under vacuum before use. Solvents were dried and deoxygenated using a Pure Process Technologies Solvent Purification System and stored over 3 Å molecular sieves. Deuterated solvents were deoxygenated with five freeze-pump-thaw cycles and stored over 3 Å molecular sieves for at least 3 days before use. $K(H_3BP^tBu_2BH_3)$, $U_2(H_3BP^tBu_2BH_3)_6$, and $Nd_2(H_3BP^tBu_2BH_3)_6$ were prepared as previously reported.[1-2] LnI_3 starting materials were used as received from Alfa Aesar or Strem Chemicals.

^{1}H NMR data were collected on a Bruker AVANCE-400 operating at 400 MHz, or a Bruker AVANCE-500 operating at 500 MHz. The ^{11}B NMR data were collected on a Bruker AVANCE-400 operating at 128 MHz or a Bruker AVANCE-500 operating at 160 MHz. ^{31}P{^{1}H} NMR data were collected on a Bruker AVANCE-500 operating at 202 MHz. Chemical shifts are reported in δ units relative to residual NMR solvent peaks (^{1}H), $BF_3{\cdot}Et_2O$ (^{11}B; δ 0.0 ppm), or 85% H_3PO_4 (^{31}P{^{1}H}; δ 0.0 ppm). Microanalytical data (CHN) were collected using an EAI CE-440 elemental analyzer at the University of Iowa's MATFab facility. IR spectra were acquired with a Thermo Scientific Nicolet iS5 in an N_2-filled glovebox as KBr pellets. Mechanochemical reactions were carried using a Form-Tech Scientific (FTS) FTS1000 Shaker Mill. The reactions were conducted in 5 mL stainless steel "SmartSnap" (hermetic seal) grinding jars from FTS using two 5 mm stainless steel balls (304 grade) for grinding.

$La_2(H_3BP^tBu_2BH_3)_6$. LaI_3 (0.201 g, 0.387 mmol) and $K(H_3BP^tBu_2BH_3)$ (0.245 g, 1.16 mmol) were loaded into a 5 mL FTS ball mill jar with two 5 mm stainless steel balls along with several drops of Et_2O. The jar was hermetically sealed, transferred to an FTS shaker mill, and

milled at 1600 rpm for 120 min. The jar was then transferred to a glovebox and opened to reveal a white residue. The contents were extracted into an 11-dram vial with 40 mL chlorobenzene and stirred for several hours. The suspension was filtered through a fine frit and evaporated to dryness under vacuum to reveal a white solid. Et_2O containing a small amount of pentane was added and gently heated until all the contents dissolved into solution (ca. 35 mL) and stored at -30˚C overnight to afford colorless plates (21 mg). Yield: 8%. Anal. Calcd for $C_{24}H_{72}B_6LaP_3$: C, 43.39; H, 11.00. Found: C, 43.59; H, 10.53 ^{1}H NMR (500 MHz, C_6D_6): δ 1.21 (d, *J* = 13 Hz, $C(CH_3)_3$), 1.26 (d, *J* = 13 Hz, $C(CH_3)_3$), 1.29 (d, *J* = 13 Hz, $C(CH_3)_3$), 1.87 (br m, BH_3). ^{11}B NMR (128 MHz, C_6D_6): δ -21.3 (br m, BH_3), -26.9 (br m, BH_3). ^{31}P{^{1}H} NMR (202 MHz, C_6D_6): δ 22.8 (br s, FWHM = 540 Hz, dimer), 2.2 (br s, FWHM = 340 Hz, dimer), 11.5 (br s, FWHM = 390 Hz, monomer). IR (cm^{-1}): 2967 (s), 2964 (s), 2947 (s), 2899 (s), 2868 (s), 2778 (w), 2709 (w), 2715 (w), 2427 (s, BH_3), 2350 (s, BH_3), 2238 (s, BH_3), 1477 (s), 1391 (s), 1367 (s), 1241 (s), 1239 (s), 1180 (s), 1130 (s), 1064 (s), 1022 (s), 934 (m), 817 (s), 794 (w), 716 (s), 623 (s), 632 (s).

La($H_3B^tBu_2PBH_3$)$_3$(thf)$_3$. LaI_3 (0.100 g, 0.192 mmol) and K($H_3BP^tBu_2BH_3$) (0.122 g, 0.576 mmol) were stirred overnight in 10 mL of chlorobenzene. The solution was filtered through a sintered glass funnel and solvent was evaporated under reduced pressure. The white residue was dissolved in the minimum amount of thf, layered with pentane, and left for two days at -30 °C to yield white needle-like crystals. Yield: 54 mg (43%) ^{1}H NMR (500 MHz, C_6D_6): δ 1.26 (d, *J* = 13 Hz, 54 H, $C(CH_3)_3$), 1.41 (m, 16 H, CH_2), 1.90 (br, 18 H, BH_3) 3.58 (m, 16 H, CH_2O). ^{11}B NMR (160 MHz, C_6D_6): δ -21.4 (br m), -27.0 (br m). IR (cm^{-1}) 2986 (s), 2965 (s), 2956 (s), 2896 (s), 2864 (s), 2775 (w), 2744 (w), 2713 (w), 2420 (s), 2344 (s), 2262 (s), 1476 (s), 1387 (m), 1363 (s), 1297 (w), 1239 (s), 1186 (s), 1129 (s), 1066 (s), 1012 (s), 936 (m), 915 (w), 860 (s), 818 (s), 749 (s), 673 (s)

$Ce_2(H_3BP^tBu_2BH_3)_6$. CeI_3 (0.100 g, 0.192 mmol) and $K(H_3BP^tBu_2BH_3)$ (0.122 g, 0.576 mmol) were transferred to a 5 mL stainless steel FTS ball milling jar followed by two 5 mm stainless steel balls and several drops of Et_2O. The reaction vessel was sealed and milled for 270 min at 1600 rpm. The vessel was transferred to a glovebox, where the resulting white paste was scraped from the jar and stirred in approximately 20 mL of Et_2O overnight. The mixture was then filtered over a fine frit and the remaining solid was washed with Et_2O (ca. 50 mL). The filtrate was evaporated to dryness under vacuum to reveal a white oily solid. The solid was suspended in boiling pentane, filtered, and evaporated to dryness under vacuum to reveal a clear oil. The oil was dissolved in thf (1 mL) and the solution was vapor diffused with pentane. After two days, large, clear blocks had formed and were recovered from the bottom of the vial (42.3 mg). Evaporating the mother liquor to dryness and repeating the vapor diffusion process with thf and pentane yielded an additional 8.2 mg of crystals. Yield: 50.5 mg (40%). Anal. Calcd for $C_{24}H_{72}B_6CeP_3$: C, 43.31; H, 10.98. Found: C, 43.37; H, 10.65. ^1H NMR (400 MHz, C_6D_6): δ 0.36 (br s, $C(CH_3)_3$), 2.00 (br d, J = 13 Hz, $C(CH_3)_3$), 2.42 (br s, $C(CH_3)_3$), 20.0 (br s, BH_3, FWHM = 260 Hz), 23.9 (br s, BH_3, FWHM = 290 Hz), 34.5 (br s, BH_3, FWHM = 290 Hz.) ^{11}B NMR (128 MHz, C_6D_6): δ -1.0 (br s, BH_3, FWHM = 320 Hz), 11.6 (br s, BH_3, FWHM = 220 Hz), 45.2 (br s, BH_3, FWHM = 240 Hz). IR (cm^{-1}): 635 (w), 670 (w), 817 (w), 934 (w), 1022 (m), 1064 (m), 1127 (m), 1182 (m), 1242 (m), 1367 (m), 1391 (w), 1477 (m), 2235 (s), 2354 (m), 2427 (s), 2867 (m), 2899 (m), 2947 (m), 2965 (m), 2968 (m).

$Pr_2(H_3BP^tBu_2BH_3)_6$. PrI_3 (0.100 g, 0.192 mmol) and $K(H_3BP^tBu_2BH_3)$ (0.122 g, 0.576 mmol) were transferred to a 5 mL stainless steel FTS ball milling jar followed by two 5 mm stainless steel balls and several drops of Et_2O. The reaction vessel was sealed, removed from the glovebox, and milled for 90 minutes at 1600 rpm. Once the milling process was complete, the

vessel was transferred to a glovebox where an opaque yellow/green paste was scraped from the jar and stirred in approximately 15 mL of Et_2O for several minutes. The mixture was filtered through a fine frit and the filtrate was evaporated to dryness under vacuum. The solid was dissolved in pentane and filtered, and the filtrate was evaporated to dryness to reveal an oil. The oil was dissolved in thf and vapor diffused with pentane. After one week at room temperature, yellowish green plate-like crystals had formed and were recovered from the bottom of the vial (99.7 mg). Evaporating the mother liquor to dryness and repeating the vapor diffusion process with thf and pentane yielded a second crop of crystals (10 mg). Yield: 110 mg (43%). Anal. Calcd for $C_{24}H_{72}B_6PrP_3{\cdot}(C_5H_{12})_{0.2}$: C, 43.48; H, 11.00. Found: C, 43.71; H, 10.65. ^{1}H NMR (400 MHz, C_6D_6): δ -0.44 (br s, $C(CH_3)_3$), 2.14 (br s, $C(CH_3)_3$), 3.10 (br d, J = 12 Hz, $C(CH_3)_3$), 53.2 (br s, BH_3, FWHM = 290 Hz), 63.9 (br s, BH_3, FWHM = 310 Hz), 85.1 (br s, BH_3, FWHM = 350 Hz). ^{11}B NMR (128 MHz, C_6D_6): δ 39.5 (br s, BH_3, FWHM = 290 Hz), 67.3 (br s, BH_3, FWHM = 200 Hz), 154.5 (br s, BH_3, FWHM = 200 Hz). IR (cm^{-1}): 818 (m), 898 (w), 935 (w), 1022 (m), 1065 (m), 1132 (w), 1182 (m), 1243 (w), 1367 (m), 1390 (w), 1476 (m), 1619 (w), 2259 (s), 2349 (m), 2428 (m), 2868 (m), 2899 (m), 2948 (m), 2964 (m), 2969 (m).

$Sm_2(H_3B^tBu_2PBH_3)_6$. $SmBr_3$ (0.100 g, 0.182 mmol) and $K(H_3BP^tBu_2BH_3)$ (0.1162 g, 0.5482 mmol) were added to a 5 mL FTS ball milling jar followed by two 5 mm stainless steel balls and several drops of Et_2O. The reaction vessel was sealed, removed from the glovebox, and milled for 90 min at 1600 rpm. Once the milling process was complete, the vessel was transferred to a glovebox where the mixture was scraped from the jar and stirred in approximately 15 mL of Et_2O for 5 minutes. The mixture was filtered through a fine frit and the filtrate was evaporated to dryness under vacuum to reveal an opaque oily solid. The solid was dissolved in pentane (ca. 40 mL), concentrated to 15 mL under vacuum, and stored at -30 ˚C to yield clear crystals after 2 days.

Yield: 82.3 mg (48%). Anal. Calcd for $C_{24}H_{72}B_6SmP_3$: C, 42.64; H, 10.81. Found: C, 42.59; H, 10.72. ^{1}H NMR (400 MHz, C_6D_6): δ -3.89 (br m, BH_3), -2.27 (br m, BH_3), -1.63 (br m, BH_3), 1.25 (m, $C(CH_3)_3$), 1.62 (br s, $C(CH_3)_3$), ^{11}B NMR (128 MHz, C_6D_6): δ -32.4 (m, BH_3), -29.0 (br m, BH_3). IR (cm^{-1}): 817 (m), 934 (w), 1021 (m), 1064 (m), 1184 (m), 1250 (m), 1368 (m), 1390 (w), 1478 (m), 2235 (s), 2355 (m), 2429 (m), 2868 (m), 2899 (m), 2947 (m), 2964 (m), 2969 (m).

2. Thermodynamic Studies

To quantify the thermodynamic parameters ΔH, ΔS, and ΔG associated with the dimer/monomer equilibrium, variable-temperature quantitative NMR spectroscopy (VT qNMR) studies were conducted in triplicate for each $M_2(H_3BP^tBu_2BH_3)_6$ complex with M = U, La, Ce, Pr, and Nd. Concentration of the C_6D_5H in C_6D_6 was determined by integration of an external standard of ferrocene with known concentration, and the C_6D_5H concentration was used to calculate absolute concentration of the dimer and monomer at each temperature. Ferrocene was purchased from Sigma Aldrich and sublimed 5 times before use as the external standard in qNMR studies, as described previously.[3] Acquisition of the ^1H qNMR external standards were carried out following known acquisition and processing procedures (temperature, flip angle, acquisition time, spectral width, and relaxation) described by Napolitano et al.[3] An 80% ethylene glycol in DMSO-D_6 standard, purchased from Cambridge Isotope Laboratories, was used to calibrate sample temperature and assess error in the temperature during the variable temperature measurements based on the known temperature-induced NMR solvent shift.[4] Concentrations of the monomer and dimer were determined by integrating the *tert*-butyl ^1H resonances for comparison to the known concentration of the C_6D_5H standard. Due to the slightly overlapping *tert*-butyl resonances for diamagnetic $La_2(H_3BP^tBu_2BH_3)_6$, integrations were determined by curve fitting the peaks in the program TopSpin (version 3.6.2).

The equilibrium constants K_{eq}, where K_{eq} = [monomer]^2/[dimer], were calculated using data collected at 300, 310, 320, 330, 340, and 345 K on triplicate samples. To ensure reversibility of monomer/dimer exchange, measurements were collected at the same temperatures from 300 to 345 K back down to 300 K (Figure S1).

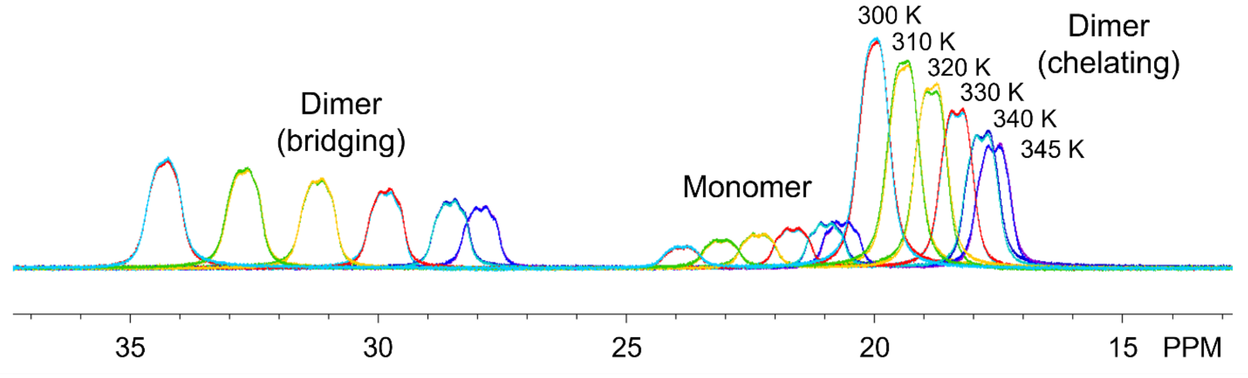

Figure S1. Overlay of the VT ^{1}H NMR spectra for the downfield monomer and dimer BH_3 resonances of $Ce(H_3BP^tBu_2BH_3)_3$ from 300 K to 345 K and back to 300 K.

Van't Hoff plots were used to quantify the enthalpy (ΔH) and entropy (ΔS) with the deoligomerization of each dimer over the given temperature range (Figure S2 – S6).[5-6] A linear regression of ln K_{eq} vs 1/T was used to generate a line to give the slope and intercept containing ΔH and ΔS, respectively (Eq 1). These values were then used to obtain ΔG according to Eq 2.

$$\ln K_{eq} = -\frac{\Delta H}{R}\frac{1}{T} + \frac{\Delta S}{R} \qquad \textbf{(1)}$$

$$\Delta G = \Delta H - T\Delta S \qquad \textbf{(2)}$$

Two methods were used to obtain the values of ΔH and ΔS and evaluate their uncertainties. In the first method, each run of each triplicate data set was plotted to obtain ΔH and ΔS values that were then averaged to obtain standard deviations for comparison (Figures S2 – S6). These values, which are provided in Table S2, are the average values and standard deviations provided in the main text.

Table S2. ΔH and ΔS values obtained by plotting ln K_{eq} vs. 1/T data from each run, as shown in Figures S2 – S6.

M	U		La		Ce		Pr		Nd	
	ΔH	ΔS	ΔH	ΔS	ΔH	ΔS	ΔH	ΔS	ΔH	ΔS
Run 1	10.3	0.017	10.1	0.018	9.6	0.016	9.5	0.017	8.6	0.015
Run 2	10.7	0.018	9.1	0.015	8.5	0.013	8.9	0.015	8.4	0.017
Run 3	10.5	0.018	9.1	0.015	8.7	0.012	8.6	0.014	9.1	0.015
avg.	10.5	0.017	9.4	0.016	8.9	0.014	9.0	0.015	8.7	0.016
std. dev.	0.2	0.001	0.6	0.002	0.5	0.002	0.4	0.001	0.4	0.001

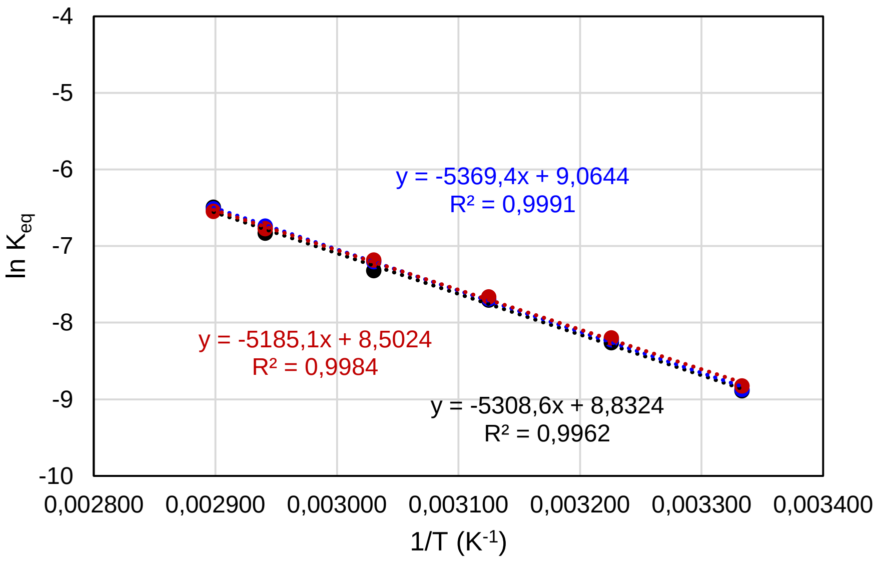

Figure S2. Van't Hoff plots from triplicate VT qNMR data collected for $U_2(H_3BP^tBu_2BH_3)_6$. Run 1 = black, run 2 = blue, and run 3 = red.

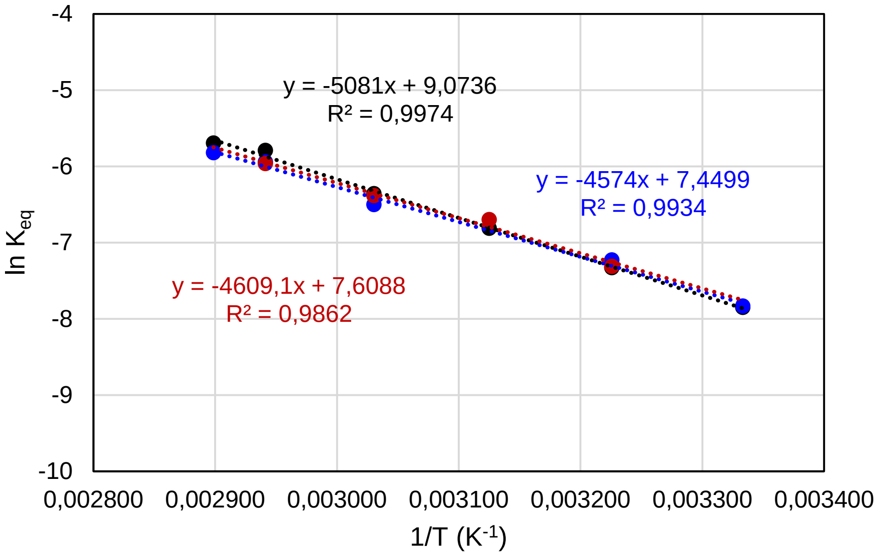

Figure S3. Van't Hoff plots from triplicate VT qNMR data collected for $La_2(H_3BP^tBu_2BH_3)_6$. Run 1 = black, run 2 = blue, and run 3 = red.

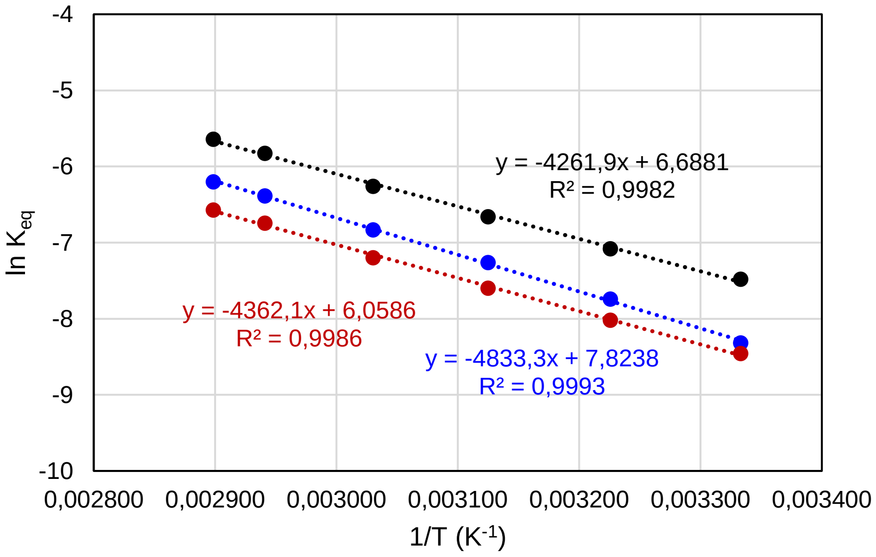

Figure S4. Van't Hoff plots from triplicate VT qNMR data collected for $Ce_2(H_3BP^tBu_2BH_3)_6$. Run 1 = black, run 2 = blue, and run 3 = red.

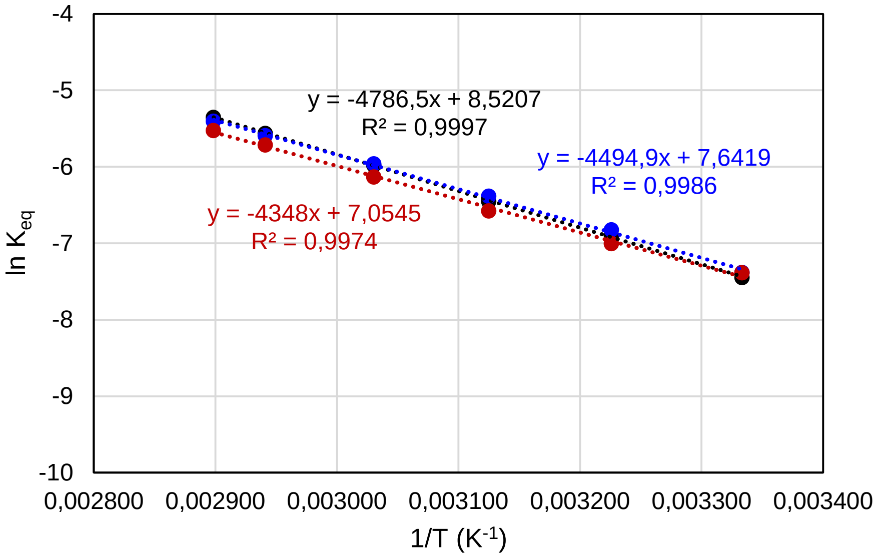

Figure S5. Van't Hoff plots from triplicate VT qNMR data collected for $Pr_2(H_3BP^tBu_2BH_3)_6$. Run 1 = black, run 2 = blue, and run 3 = red.

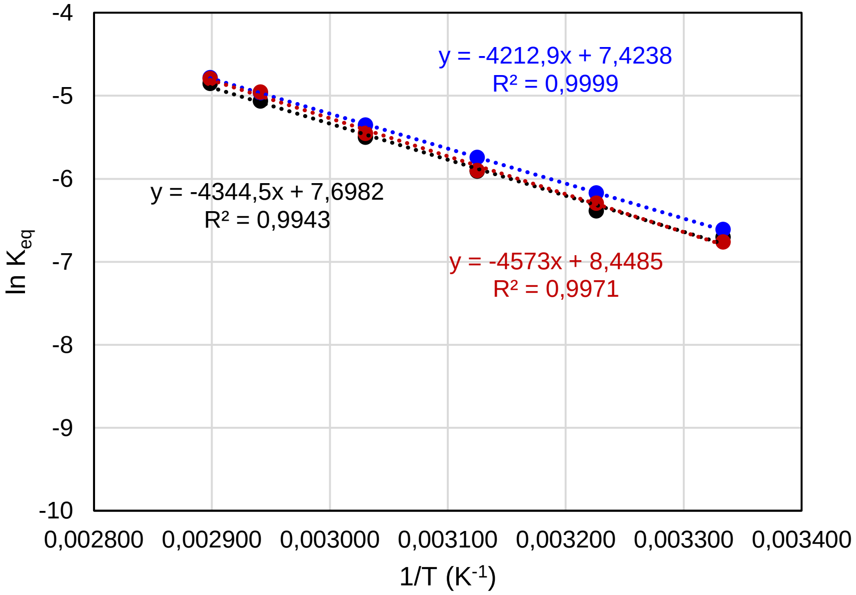

Figure S6. Van't Hoff plots from triplicate VT qNMR data collected for $Nd_2(H_3BP^tBu_2BH_3)_6$. Run 1 = black, run 2 = blue, and run 3 = red.

The second method used is the error propagation method described by Xue and coworkers, as shown in Eqs 3 and 4.[7-9] The drawback of this method is that it is known to give relatively large uncertainties in the values of ΔH and ΔS when the temperature range of the data collection (ΔT) is limited because of ΔT^2 and ΔT^4 in the denominator of both equations. This is true of the data described in this report; the equilibrium data could only be collected over a relatively short temperature range (ΔT = 45 K) because of the limited solubility of the complexes in cooled solutions and concerns about the reduced stability of the complexes at higher temperatures. We view the uncertainties obtained using this method as beingly overly conservative given the relatively high reproducibility of the data, as shown in Table S2. This is especially true with respect to the slope of the linear regressions where ΔH is derived. Nevertheless, we wish to provide the uncertainty analysis using Eqs 3 and 4 to show how the values compare.

$$(\sigma\Delta H)^2 = \frac{\left(\frac{\sigma T}{T}\right)^2 R^2\left(T_{max}{}^2 T_{min}{}^4 + T_{min}{}^2 T_{max}{}^4\right)\left[\ln\left(\frac{K_{eq(max)}}{K_{eq(min)}}\right)\right]^2}{\Delta T^4} + \frac{2R^2\left(T_{min}{}^2 T_{max}{}^2\right)\left(\frac{\sigma K_{eq}}{K_{eq}}\right)^2}{\Delta T^2} \quad \textbf{(3)}$$

$$(\sigma\Delta S)^2 = \frac{2R^2 T_{min}{}^2 T_{max}{}^2\left[\ln\left(\frac{K_{eq(max)}}{K_{eq(min)}}\right)\right]^2\left(\frac{\sigma T}{T}\right)^2}{\Delta T^4} + \frac{R^2\left(T_{max}{}^2 + T_{min}{}^2\right)\left(\frac{\sigma K_{eq}}{K_{eq}}\right)^2}{\Delta T^2} \quad \textbf{(4)}$$

Determining ΔH and ΔS using this second method requires plotting the natural log of the averaged K_{eq} values obtained at each temperature vs. 1/T (Figure S7). As expected, the ΔH and ΔS values obtained using this method are almost identical to those obtained using the first method (Tables S2 and S3). The uncertainties were then obtained using Eqs 3 and 4 and the following key variables. The uncertainty in temperature (σT/T) was 1 K. The average K_{eq} and standard deviations ($\sigma K_{eq(ran)}$) determined at each temperature were used to calculate the random uncertainty ($\sigma K_{eq(ran)}/K_{eq}$) in the data for that temperature. The largest random uncertainty obtained for each

data set was the value that was combined with the systematic uncertainty ($\sigma K_{eq(sys)}/K_{eq}$), which is typically estimated at 5%, to obtain the total uncertainty $\sigma K_{eq}/K_{eq}$ according to Eq 5.

$$\sigma K_{eq}/K_{eq} = [(\sigma K_{eq(sys)}/K_{eq})^2 + (\sigma K_{eq(ran)}/K_{eq})^2]^{1/2} \qquad \textbf{(5)}$$

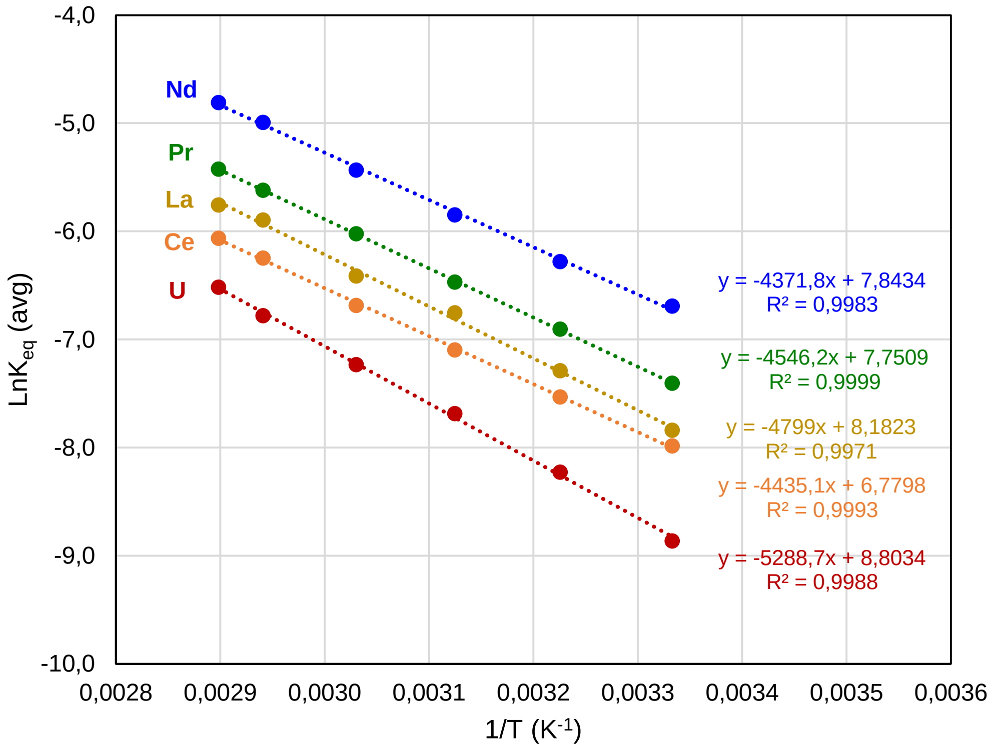

Figure S7. Van't Hoff plots obtained for each complex using the natural log of averaged K_{eq} values at each temperature.

The $\sigma K_{eq}/K_{eq}$ values determined for all the complexes except M = Ce (see below) ranged from 9 – 12%, which are comparable to the 13 – 15% reported by Xue and coworkers for $\sigma K_{eq}/K_{eq}$ values determined for Van't Hoff data collected over a similarly small temperature ranges (ΔT = 30 – 40 K). However, the data collected for the Ce complex showed much larger uncertainties via the analysis because of greater differences in the values obtained for the y-intercepts, as shown in Figure S4. It is not clear why there was greater variance in the y-intercept data obtained for the Ce

complex. We repeated several additional VT runs and saw similar variability that did not improve the statistics on the y-intercept.

Table S3. Average ΔH ($kcal\cdot mol^{-1}$) and ΔS ($kcal\cdot mol^{-1}\cdot K^{-1}$) values obtained using the plot in Figure S7 and the error propagation method of Xue and coworkers (Eqs 3 and 4).

M	U		La		Ce		Pr		Nd	
	ΔH	ΔS	ΔH	ΔS	ΔH	ΔS	ΔH	ΔS	ΔH	ΔS
avg.	10.5	0.017	9.5	0.018	8.8	0.013	9.0	0.015	8.7	0.016
uncertainty	0.7	0.002	0.8	0.003	3.7	0.012	0.8	0.002	0.8	0.003

The larger run-to-run range of y-intercept values obtained for M = Ce, which describes the y-offset of the line, resulted in a total uncertainty of $\sigma K_{eq}/K_{eq}$ = 57% due to the large standard deviation in K_{eq}. When combined with the relatively small ΔT, Eqs. 3 and 4 yield values of ΔH = 8.8 ± 3.7 $kcal\cdot mol^{-1}$ and ΔS = 0.013 ± 0.012 $kcal/mol^{-1}\cdot K^{-1}$. These uncertainties are unreasonably large when compared with the values of ΔH = 8.9 ± 0.5 $kcal\cdot mol^{-1}$ and ΔS = 0.014 ± 0.002 $kcal\cdot mol^{-1}\cdot K^{-1}$ in Table S2, especially considering that the consistency in the slopes of the lines where ΔH is determined. The uncertainties in ΔH for the other complexes are more reasonable at 0.7 – 0.8 $kcal\cdot mol^{-1}$ and slightly larger than those obtained using the standard deviations due to the dependence on the small ΔT. The values used in Eqs. 3 and 4 and Figure S7 are provided in Table S3 and S4 for comparison to the standard deviations in Table S2.

Table S4. Average K_{eq}, $\sigma K_{eq(ran)}$, $\sigma K_{eq(ran)}/K_{eq}$, $\sigma K_{eq}/K_{eq}$, and LnKeq determined for each complex at each temperature.

	Temp (K)	Avg. K_{eq}	Std. Dev. ($\sigma K_{eq(ran)}$)	LnK_{eq}	$\sigma K_{eq(ran)}/K_{eq}$	$\sigma K_{eq}/K_{eq}$
M = U	300	1.42E-04	4.56E-06	-8.86	3%	6%
	310	2.67E-04	8.52E-06	-8.23	3%	6%
	320	4.60E-04	9.82E-06	-7.68	2%	5%
	330	7.22E-04	5.26E-05	-7.23	7%	9%
	340	1.13E-03	5.43E-05	-6.78	5%	7%
	345	1.48E-03	3.89E-05	-6.52	3%	6%
M = La	300	3.93E-04[a]	4.66E-06	-7.84	-[a]	-[a]
	310	6.83E-04	3.70E-05	-7.29	5%	7%
	320	1.17E-03	9.28E-05	-6.75	11%	12%
	330	1.64E-03	1.23E-04	-6.41	8%	9%
	340	2.75E-03	2.64E-04	-5.90	10%	11%
	345	3.16E-03[a]	2.76E-04	-5.76	-[a]	-[a]
M = Ce	300	3.40E-04	1.94E-04	-7.99	57%	57%
	310	5.36E-04	2.71E-04	-7.53	51%	51%
	320	8.28E-04	4.04E-04	-7.10	49%	49%
	330	1.25E-03	5.99E-04	-6.69	48%	48%
	340	1.94E-03	9.13E-04	-6.25	47%	47%
	345	2.32E-03	1.10E-03	-6.06	47%	48%
M = Pr	300	6.09E-04	2.19E-05	-7.40	4%	6%
	310	1.00E-03	9.16E-05	-6.90	9%	10%
	320	1.55E-03	1.49E-04	-6.47	10%	11%
	330	2.43E-03	2.26E-04	-6.02	9%	11%
	340	3.62E-03	2.85E-04	-5.62	8%	9%
	345	4.40E-03	3.87E-04	-5.43	9%	10%
M = Nd	300	1.24E-03	9.70E-05	-6.69	8%	9%
	310	1.87E-03	2.04E-04	-6.28	11%	12%
	320	2.88E-03	2.75E-04	-5.85	10%	11%
	330	4.37E-03	3.26E-04	-5.43	7%	9%
	340	6.78E-03	4.09E-04	-4.99	6%	8%
	345	8.16E-03	3.12E-04	-4.81	4%	6%

[a]The ^{1}H NMR resonances at 300 K and 345 K from run 3 were not resolved enough to obtain reliable curve fits for integration, so only data from run 1 and 2 were averaged for these two temperatures.

3. Crystallographic Studies

$M_2(H_3BP^tBu_2BH_3)_6$ crystals suitable for single crystal X-ray analysis were grown from either thf and pentane (M = Ce, Pr), Et_2O and pentane (M = La), or pentane (M = Sm) and mounted on a MiTeGen micromount with ParatoneN oil. Crystals of $La(H_3BP^tBu_2BH_3)_3(thf)_3$ were grown from thf and pentane. Crystallographic data were collected from two diffractometers, the first, a Bruker Nonius Kappa ApexII, equipped with a charge-coupled-device (CCD) detector and cooled to 150 K using an Oxford Cryostreams 700 low temperature device. The second diffractometer was a Bruker D8 Venture Duo, equipped with a Bruker photon III detector and cooled to 150 K using an Oxford Cryostream 700 low temperature device. Both instruments were equipped with a graphite monchromatized MoK_α radiation (λ = 0.71073 Å). A hemisphere of data was collected using phi and omega scans. The data were corrected for absorption using redundant reflections and the SADABS program. Structures were solved with intrinsic phasing (SHELXT)[10] and least square refinement (SHELXL),[11-12] which confirmed the positions of all non-hydrogen atoms. All hydrogen atom positions were idealized and were allowed to ride on the attached carbon and boron atoms. B-H distances were fixed at 1.20 Å. Structure solution and refinement were performed with Olex2.[13] Publication figures were made using Mercury version 4.3.1 or Olex2.[13-14]

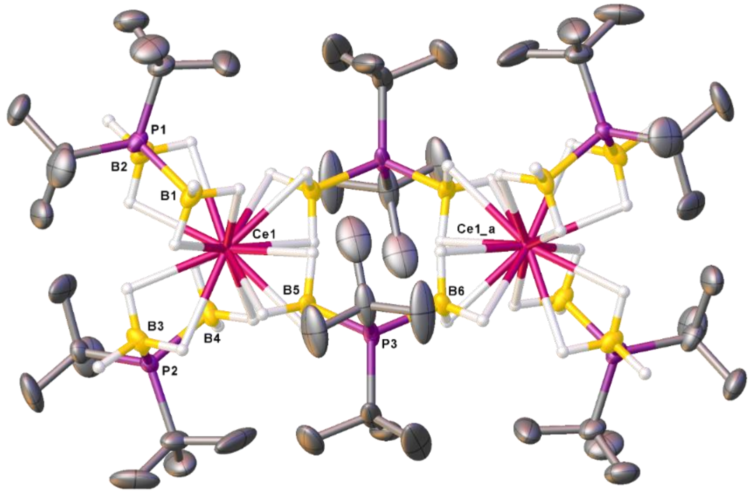

Figure S8. Molecular structure of $Ce_2(H_3BP^tBu_2BH_3)_6$ with thermal ellipsoids at 50% probability. Hydrogen atoms attached to carbon and co-crystallized pentane were omitted from the figure for clarity.

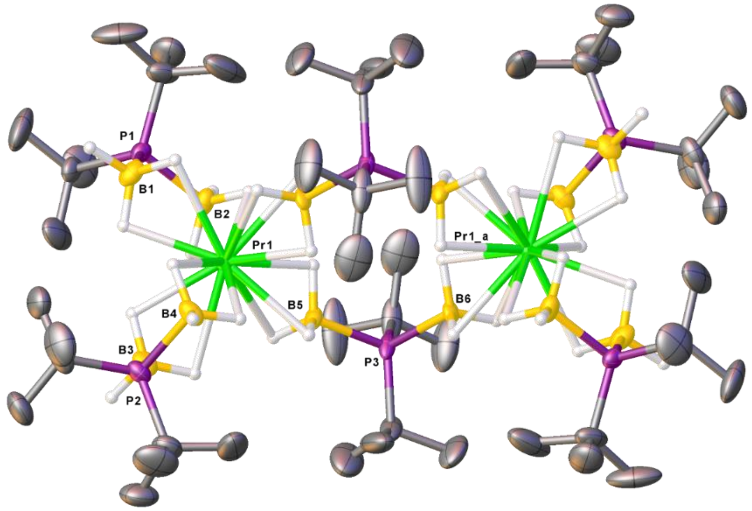

Figure S9. Molecular structure of $Pr_2(H_3BP^tBu_2BH_3)_6$ with thermal ellipsoids at 50% probability. Hydrogen atoms attached to carbon and co-crystallized pentane were omitted from the figure for clarity.

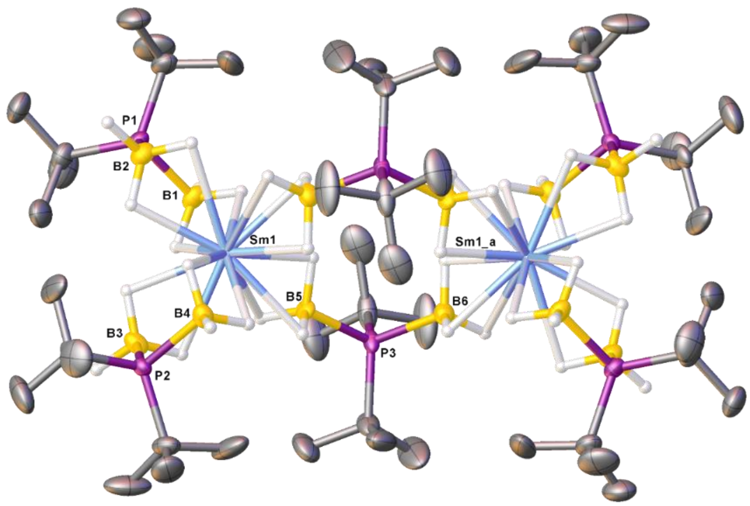

Figure S10. Molecular structure of $Sm_2(H_3BP^tBu_2BH_3)_6$ with thermal ellipsoids at 50% probability. Hydrogen atoms attached to carbon and co-crystallized pentane were omitted from the figure for clarity.

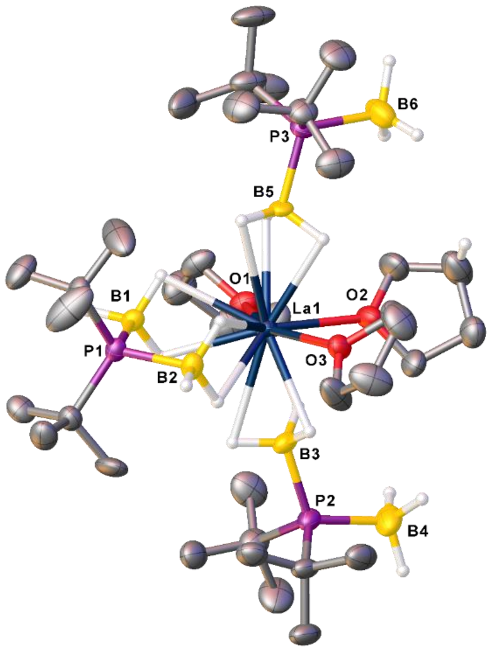

Figure S11. Molecular structure of $La(H_3BP^tBu_2BH_3)_3(thf)_3$ with thermal ellipsoids at 50% probability. Hydrogen atoms attached to carbon and disordered components were omitted from the figure for clarity.

Table S5. Single-crystal X-ray diffraction data collected for $M_2(H_3BP^tBu_2BH_3)_6$ complexes where M = La, Ce, Pr, and Sm.

**M**	**La**	**Ce**	**Pr**	**Sm**
Identifier	Dal21_51	Dal18_68	Dal18_46	Dal18_47
Formula	$C_{48}H_{144}B_{12}La_2P_6{\cdot}(C_5H_{12})_2$	$C_{48}H_{144}B_{12}Ce_2P_6{\cdot}(C_5H_{12})_2$	$C_{48}H_{144}B_{12}Pr_2P_6{\cdot}(C_5H_{12})_2$	$C_{48}H_{144}B_{12}Sm_2P_6{\cdot}(C_5H_{12})_2$
FW (g mol^{-1})	1459.27	1461.69	1463.27	1482.15
crystal system	monoclinic	monoclinic	monoclinic	monoclinic
space group	$P2_1/n$	$P2_1/n$	$P2_1/n$	$P2_1/n$
a (Å)	15.0535(15)	14.9970(15)	15.0091(15)	14.9800(15)
b (Å)	11.9472(12)	11.9398(12)	11.9250(12)	11.8904(12)
c (Å)	24.703(2)	24.654(2)	24.681(2)	24.675(2)
α (deg)	90	90	90	90
β (deg)	90.449(5)	90.228(5)	90.133(5)	90.302(5)
γ (deg)	90	90	90	90
volume $(Å)^3$	4442.6(7)	4414.5(7)	4417.5(7)	4395.0(7)
Z	2	2	2	2
ρ_{calc} (g cm^{-3})	1.091	1.100	1.100	1.120
μ (mm^{-1})	1.085	1.156	1.227	1.461
F (000)	1560	1564	1568	1580
θ range (deg)	2.176/28.341	2.716/27.959	1.586/27.133	1.587/27.159
R (int)	0.0417	0.0421	0.0220	0.0549
data/restraints/parameters	11020/0/396	10482/0/378	9593/33/378	9588/6/378
GOF	1.118	1.063	1.251	1.032
R_1 $[I > 2\sigma(I)]^a$	0.0358	0.0353	0.0444	0.0334
wR_2 (all data)^b	0.0792	0.0782	0.985	0.0711
Largest Peak/Hole (e · $Å^{-3}$)	1.481/-0.563	0.705/-0.704	1.353/-0.776	0.699/-0.474
Temp (K)	150(2)	150(2)	150(2)	150(2)

$^aR_1 = \Sigma\ |F_o| - |F_c|\ |\ /\ |\ |\Sigma|F_o|$ for reflections with $F_o^2 > 2\sigma(F_o^2)$.

$^bwR_2 = [\Sigma w(F_o^2 - F_c^2)^2 / \Sigma(F_o^2)^2]^{1/2}$ for all reflections.

Table S6. Average chelating and bridging M-B distances (Å) for $M_2(H_3BP^tBu_2BH_3)_6$ complexes where M = U, La, Ce, Pr, Nd, and Sm (values plotted in Figure 2).

	Metal	Average	Stdev
Chelating	U	2.920	0.028
	La	2.932	0.024
	Ce	2.910	0.030
	Pr	2.899	0.031
	Nd	2.886	0.039
	Sm	2.866	0.048
Bridging	U	2.692	0.003
	La	2.740	0.006
	Ce	2.714	0.005
	Pr	2.692	0.006
	Nd	2.670	0.006
	Sm	2.650	0.011

4. Computational Studies

Computational Details. The ^{t}Bu-PDB complexes of La, Ce, Pr, Nd, and U were optimized, and harmonic vibrational frequencies were computed in the gas phase to obtain free energies by means of density functional theory (DFT). Geometries were optimized with the TPSS functional with Grimme's D3 corrections (TPSS-D3) and M06-L functional. The resolution of identity approximation was used for integral evaluation.[15-19] Because diffraction experiments are only available for the dimeric species, a conformational search was performed for the La monomer using the Conformer-Rotamer Ensemble Sampling Tool (CREST) algorithm in the xtb program.[20] Single point computations were performed using the TPSS, TPSS-D3, M06-L, M06, TPSSh, and TPSSh-D3 functionals. All single point calculations include the conductor-like screening model (COSMO)[21] to account for solvation using a dielectric constant of 2.274 for benzene. The def2-

TZVP basis set is used on all atoms except for U where the def-TZVP basis was empolyed.[22-29] The SCF energy was converged to 10^{-7} a.u. and the Cartesian gradient was converged to 10^{-4} a.u. Some of the species have very small (<15 cm^{-1}) imaginary modes associated with methyl rotations. These did not impact the computed free energies because the quasiharmonic correction suggested by Cramer and Truhlar is used for all thermochemistry data in which normal modes less than 100 cm^{-1} are replaced with 100 cm^{-1}.[30] Free energies are computed at 298.15 K and assuming a concentration of 1 M for all reactants and products. The TPSS-D3 calculations (single point and optimization with the same functional) were further analyzed by computing CM5 charges, the topological analysis of the electron density with Bader's Atoms in Molecules (QTAIM) and delocalization index (δ) as implemented in the MultiWFN 3.8 software package.[31-32] Thermochemistry results are not sensitive to the geometry (TPSS-D3 or M06-L). Hybrid functionals performed similar to their local counterpart and the inclusion of dispersion, either using D3 or by using the Minnesota functionals is important. However, TPSS-D3 and TPSSh-D3 are in better agreement with experimental free energies, while M06-L and M06 are in better agreement with experimental enthalpies (Table S7-S11). Since the same trends emerge with all functionals, we include the TPSS-D3 results (and additional analysis at the same level) in the manuscript.

DFT Results.

Table S7. The thermochemical data at 298.15K for the functional included in the manuscript. Specifically, TPSS-D3 electronic energies, enthalpies, entropies, and free energies for reaction $M_2(H_3BP^tBu_2BH_3)_6 \rightarrow 2\ M(H_3BP^tBu_2BH_3)_3$. Free energies have been computed assuming a concentration of 1 M for all species and benzene as the solvent. The geometry optimization and single point calculation are performed with the same functional.

M	ΔE (kcal/mol)	ΔH (kcal/mol)	ΔS (kcal/mol K)	ΔG (kcal/mol)
La	20.1	18.6	0.053	4.8
Ce	18.9	18.0	0.053	4.1
Pr	20.2	18.6	0.055	4.2
Nd	18.8	17.4	0.053	3.6
U	21.9	20.6	0.054	6.3

Table S8. The reaction enthalpy computed treating the electronic energy using six functionals on the TPSS-D3 geometry for the reaction $M_2(H_3BP^tBu_2BH_3)_6 \rightarrow 2\ M(H_3BP^tBu_2BH_3)_3$ with benzene as the solvent. Thermal corrections are treated at the TPSS-D3 level.

M	TPSS	TPSSh	TPSS-D3	TPSSh-D3	M06-L	M06	Exp.
U	6.4	6.7	20.6	21.3	16.4	16.0	10.5 ± 0.2
La	3.6	4.1	18.6	19.4	16.2	15.2	9.4 ± 0.6
Ce	-1.9	-1.2	18.0	18.8	13.7	13.3	8.9 ± 0.5
Pr	-0.7	-0.04	18.6	19.4	14.6	12.2	9.0 ± 0.4
Nd	-0.3	0.2	17.4	19.5	13.6	11.5	8.7 ± 0.4

Table S9. The reaction free energy computed treating the electronic energy using six functionals on the TPSS-D3 geometry for the reaction $M_2(H_3BP^tBu_2BH_3)_6 \rightarrow 2\ M(H_3BP^tBu_2BH_3)_3$ with benzene as the solvent. Thermal corrections are treated at the TPSS-D3 level.

M	TPSS	TPSSh	TPSS-D3	TPSSh-D3	M06-L	M06	Exp.
U	-7.9	-7.6	6.3	7.0	2.1	1.7	5.3 ± 0.2
La	-10.2	-9.7	4.8	5.5	2.4	1.4	4.6 ± 0.6
Ce	-15.8	-15.1	4.1	4.9	-0.2	-0.6	4.7 ± 0.5
Pr	-15.1	-14.4	4.2	5.0	0.2	-2.1	4.4 ± 0.4
Nd	-14.1	-13.6	3.6	5.6	-0.2	-2.4	4.0 ± 0.4

Table S10. The reaction enthalpy computed treating the electronic energy using six functionals on the M06-L geometry for the reaction $M_2(H_3BP^tBu_2BH_3)_6 \rightarrow 2\ M(H_3BP^tBu_2BH_3)_3$ with benzene as the solvent. Thermal corrections are treated at the M06-L level.

M	TPSS	TPSSh	TPSS-D3	TPSSh-D3	M06-L	M06	Exp.
U	7.0	7.3	20.3	21.0	17.3	17.8	10.5 ± 0.2
La	3.5	3.9	19.0	19.6	16.6	15.6	9.4 ± 0.6
Ce	-0.03	0.5	18.4	19.1	15.3	14.9	8.9 ± 0.5
Pr	3.2	3.7	18.5	19.3	17.0	13.9	9.0 ± 0.4
Nd	4.1	4.0	19.5	19.7	14.1	13.6	8.7 ± 0.4

Table S11. The reaction free energy computed treating the electronic energy using six functionals on the M06-L geometry for the reaction $M_2(H_3BP^tBu_2BH_3)_6 \rightarrow 2\ M(H_3BP^tBu_2BH_3)_3$ with benzene as the solvent. Thermal corrections are treated at the M06-L level.

M	TPSS	TPSSh	TPSS-D3	TPSSh-D3	M06-L	M06	Exp.
U	-6.7	-6.3	6.6	7.3	3.7	4.1	5.3 ± 0.2
La	-10.5	-10.1	5.1	5.6	2.7	1.7	4.6 ± 0.6
Ce	-14.1	-13.6	4.3	5.0	1.3	0.8	4.7 ± 0.5
Pr	-10.3	-9.8	5.0	5.8	3.5	0.4	4.4 ± 0.4
Nd	-9.4	-9.5	6.0	6.2	0.6	0.05	4.0 ± 0.4

DFT Geometries. The geometries optimized at the RI-TPSS-D3/def2-TZVP(def-TZVP for U) and RI-M06-L/def2-TZVP(def-TZVP for U) levels of theory are in good agreement with structures from diffraction experiments, when available. The bond distances of chelating M-B distances followed the same trend as the experimental results. For example, M-B distances with TPSS-D3 decrease from La to Nd with decreasing ionic radius, while the U-B distances fall in between La and Ce (Table S13). In the case of bridging ligands, the U-B distances were significantly shorter than for La, Ce, Pr, and Nd, which is in good agreement with those seen experimentally. The conformer search for $La(H_3BP^tBu_2BH_3)_3$ produced 18 conformers; each were optimized at the RI-TPSS-D3/def2-TZVP level of theory. All conformers and relative energies are reported (Table S12). M06-L results are in the subsequent tables.

Table S12. The energy and RMSD of the La($H_3BP^tBu_2BH_3$)$_3$ conformers optimized with RI-TPSS-D3/def2-TZVP level of theory.

**Conformers**	**Energy kcal/mol**	**RMSD**	**Conformers**	**Energy kcal/mol**	**RMSD**
**0**	**0.0**	**-**			
1	6.0	5.553	10	7.0	5.549
2	6.3	5.594	11	6.3	5.650
3	6.5	6.110	12	6.7	5.867
4	6.6	5.760	13	6.7	5.546
5	7.0	5.737	14	6.7	5.976
6	7.0	5.878	15	6.9	5.848
7	7.0	5.724	16	6.9	5.838
8	6.3	5.627	17	6.6	5.83
9	6.6	5.731	18	6.6	5.537

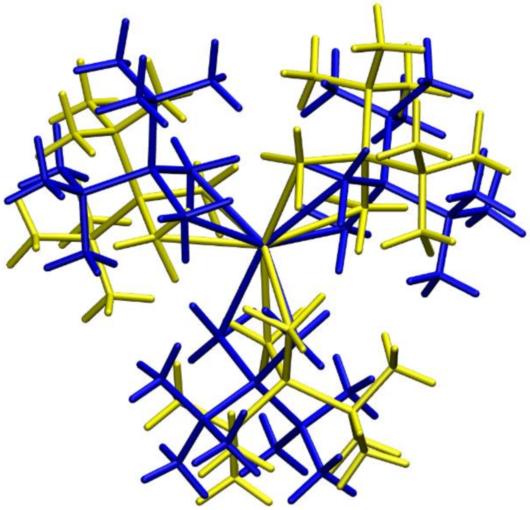

Figure S12. The most stable conformer (blue) and conformer 1 (yellow) of the La($H_3BP^tBu_2BH_3$)$_3$ structures are shown.

Table S13. Selected bond distances (Å) and angles (°) of dimers from the RI-TPSS-D3/def2-TZVP (def-TZVP for U) on optimized structures of the dimers.

dimer	M-B (Å) Chelating	M-B (Å) Bridging	B-P-B (°) Chelating	B-P-B (°) Bridging
U	2.862	2.617	103.7	108.3
La	2.906	2.728	108.9	110.2
Ce	2.832	2.756	109.9	117.7
Pr	2.829	2.723	107.7	117.3
Nd	2.822	2.696	107.2	116.3

Table S14. Selected bond distances (Å) and angles (°) from the RI-TPSS-D3/def2-TZVP (def-TZVP for U) on optimized structures of the monomers.

Monomer	M-B (Å)	B-P-B (°)
U	2.806	107.8
La	2.870	110.2
Ce	2.814	110.5
Pr	2.804	109.3
Nd	2.794	108.2

Table S15. Selected bond distances (Å) and angles (°) of dimers from the RI-M06-L/def2-TZVP (def-TZVP for U) on optimized structures of the dimers.

dimer	M-B (Å) Chelating	M-B (Å) Bridging	B-P-B (°) Chelating	B-P-B (°) Bridging
U	2.910	2.692	106.8	109.0
La	2.925	2.798	110.6	113.3
Ce	2.871	2.796	110.8	118.3
Pr	2.874	2.712	109.2	111.3
Nd	2.870	2.685	107.7	111.1

Table S16. Selected bond distances (Å) and angles (°) from the RI-M06-L/def2-TZVP (def-TZVP for U) on optimized structures of the monomers.

Monomer	M-B (Å)	B-P-B (°)
U	2.852	109.9
La	2.895	111.4
Ce	2.848	111.6
Pr	2.834	110.6
Nd	2.821	109.6

Table S17. CM5 charge for metals and boron calculated on the ground state TPSS-D3 structure.

Dimer	M Charge	Chelating B Charge	Bridging B Charge
U	1.04	-0.49	-0.55
La	1.25	-0.52	-0.56
Ce	1.06	-0.50	-0.52
Pr	1.25	-0.52	-0.55
Nd	1.25	-0.52	-0.56

Quantum Theory of Atoms in Molecules (QTAIM). To further understand the nature of the chemical bonding in the chelating and bridging M-B bonds, we performed a topological analysis of the electron density using the quantum theory of atoms in molecules (QTAIM) developed by Bader using the RI-TPSS-D3 geometry and single point calculation. In QTAIM, a chemical bond is present if a line of locally maximum electron density joins neighboring atoms. A bond critical point (BCP) is a point along the bond path where the electron density reaches a minimum. At a BCP, the gradient (ρ) of the electron density is zero and the Laplacian of the electron density, $\nabla^2(\rho)$, could be positive or negative. A positive Laplacian means a local depletion of charge while a negative value corresponds to a local concentration of charge. In a covalent bond, the Laplacian should be negative since it is a sign of shared interaction of electron density between two linked atoms. A closed-shell interaction is associated with a positive Laplacian and these types of bonds generally are not considered covalent due to depletion of charge at the location of the BCP. The total electronic energy density, E(r), at the BCP is defined as the sum of the Lagrangian kinetic energy, G(r), and the potential energy density, V(r).

The ratio of the absolute potential energy and Lagrangian kinetic energy (|V(r)|/G(r)) at bond critical point is the indicator of type of bonding. For pure closed shell interactions |V(r)|/G(r)) < 1 which indicate fully ionic or van der Waals interactions. For shared shell interaction |V(r)|/G(r) > 2 which is fully covalent. If the value of |V(r)|/G(r)) is between 1 and 2, that indicates partial covalency. We also report the bond degree (BD), defined as E(r)/ρ, that gives a measure of the degree of covalency in these bonds. More negative values of BD suggest a greater covalent interaction.[32-34]

We obtained bond critical points for all chelating and bridging U-B and La-B bonds. However, for the Ce, Pr and Nd dimers, we did not find all BCPs despite searching from several

starting points (*i.e.*, using the various options available in MultiWFN). The properties of individual BCPs are shown in Tables S19 – S23. The average values of these properties are given in Table S18. The positive Laplacian at all BCPs indicates that these types of bonds are predominantly ionic; however, the negative energy density and the value of |V(r)|/G(r) falling in between 1 and 2 support that these bonds have some partial covalent character. The total electron density (ρ) at the chelating M-B bonds is lower in all lanthanides and uranium than the bridging ones. However, for the U-B bonds the electron density is higher than the lanthanides in both bridging and chelating ligands (Figure 4, Figure S13). This indicates that U-B bonds have more covalent character than the lanthanides. This is also supported by the bond degree parameter which is more negative for U-B bonds.

Finally, we compute the delocalization index (δ), another tool to assess covalent contributions to bonding, that quantifies the number of electrons shared by the two atoms. The higher the value, the more covalent the bond.[35-37] In these complexes, the highest delocalization index was observed for U-B bonds in the bridging ligands supporting the conclusion of increased covalent character (Figure S14, Table S24). In the lanthanide M-B bonds, the delocalization index is almost the same regardless of metal, which is consistent with bonding that does not change much across the series.

Table S18. Average properties at the bond critical points for U, La, Ce, Pr and Nd dimers. All values are expressed in atomic units.

Ligand type	Bonds	$\nabla^2(\rho)$	E(r)	ρ	|V(r)|/G(r)	BD = E(r)/ρ
Bridging	U-B	0.10440	-0.00754	0.04401	1.22420	-0.17140
	La-B	0.09288	-0.00237	0.03409	1.09309	-0.06956
	Ce-B	0.09751	-0.00336	0.03898	1.12111	-0.08618
	Pr-B	0.10328	-0.00383	0.04013	1.12928	-0.09553
	Nd-B	0.10681	-0.00398	0.04012	1.12965	-0.09914
Chelating	U-B	0.10008	-0.00280	0.03697	1.10054	-0.07566
	La-B	0.08123	-0.00074	0.03012	1.03529	-0.02456
	Ce-B	0.08518	-0.00175	0.03424	1.07586	-0.05105
	Pr-B	0.08888	-0.00166	0.03438	1.06968	-0.04841
	Nd-B	0.05577	-0.00029	0.02175	1.02067	-0.01353

Table S19. Properties at the bond critical points for U dimer. All values are expressed in atomic units. Atom labels are shown in Figure S15.

Ligand type	Bonds	$\nabla^2(\rho)$	E(r)	ρ	G(r)	V(r)
Bridging	212U-180B	0.10372	-0.00931	0.04665	0.03524	-0.04455
	212U-187B	0.10516	-0.00584	0.04140	0.03213	-0.03796
	211U-188B	0.10485	-0.00576	0.04124	0.03197	-0.03772
	211U-179B	0.10386	-0.00927	0.04674	0.03524	-0.04451
Chelating	212U-164B	0.09982	-0.00258	0.03633	0.02753	-0.03011
	212U-172B	0.09823	-0.00308	0.03746	0.02764	-0.03071
	212U-156B	0.09995	-0.00257	0.03629	0.02756	-0.03013
	212U-148B	0.10244	-0.00295	0.03779	0.02856	-0.03152
	211U-171B	0.09813	-0.00308	0.03747	0.02761	-0.03070
	211U-163B	0.09979	-0.00257	0.03631	0.02752	-0.03009
	211U-155B	0.09981	-0.00258	0.03629	0.02753	-0.03011
	211U-147B	0.10247	-0.00296	0.03780	0.02857	-0.03153

Table S20. Properties at the bond critical points for La dimer. All values are expressed in atomic units. Atom labels are shown in Figure S15.

Ligand type	Bonds	$\nabla^2(\rho)$	E(r)	ρ	G(r)	V(r)
Bridging	212La-180B	0.09047	-0.00189	0.03432	0.02439	-0.02628
	212La-187B	0.09527	-0.00285	0.03389	0.02656	-0.02941
	211La-188B	0.09519	-0.00283	0.03386	0.02653	-0.02936
	211La-179B	0.09057	-0.00191	0.03430	0.02443	-0.02634
Chelating	212La-164B	0.08037	-0.00077	0.03016	0.02077	-0.02153
	212La-172B	0.08387	-0.00085	0.03120	0.02171	-0.02256
	212La-156B	0.07973	-0.00073	0.02990	0.02057	-0.02130
	212La-148B	0.08318	-0.00078	0.03073	0.02148	-0.02225
	211La-171B	0.07957	-0.00054	0.02822	0.02036	-0.02090
	211La-163B	0.08024	-0.00076	0.03011	0.02072	-0.02148
	211La-155B	0.07987	-0.00074	0.02996	0.02062	-0.02135
	211La-147B	0.08296	-0.00077	0.03066	0.02141	-0.02218

Table S21. Properties at the bond critical points for Ce dimer. All values are expressed in atomic units. Atom labels are shown in Figure S15.

Ligand type	Bonds	$\nabla^2(\rho)$	E(r)	ρ	G(r)	V(r)
Bridging	212Ce-187B	0.09747	-0.00335	0.03896	0.02772	-0.03107
	211Ce-188B	0.09755	-0.00337	0.03900	0.02775	-0.03112
Chelating	212Ce-164B	0.08519	-0.00175	0.03418	0.02305	-0.02480
	212Ce-172B	0.08768	-0.00196	0.03539	0.02388	-0.02585
	212Ce-156B	0.08266	-0.00153	0.03316	0.02220	-0.02373
	211Ce-171B	0.08766	-0.00196	0.03537	0.02387	-0.02583
	211Ce-163B	0.08519	-0.00175	0.03418	0.02305	-0.02480
	211Ce-155B	0.08271	-0.00153	0.03316	0.02221	-0.02373

Table S22. Properties at the bond critical points for Pr dimer. All values are expressed in atomic units. Atom labels are shown in Figure S15.

Ligand type	Bonds	$\nabla^2(\rho)$	E(r)	ρ	G(r)	V(r)
Bridging	212Pr-187B	0.10332	-0.00384	0.04014	0.02967	-0.03352
	211Pr-188B	0.10324	-0.00383	0.04012	0.02964	-0.03346
Chelating	212Pr-164B	0.08925	-0.00161	0.03418	0.02393	-0.02554
	212Pr-148B	0.08822	-0.00177	0.03482	0.02383	-0.02560
	211Pr-163B	0.08916	-0.00161	0.03414	0.02390	-0.02550

Table S23. Properties at the bond critical points for Nd dimer. All values are expressed in atomic units. Atom labels are shown in Figure S15.

Ligand type	Bonds	$\nabla^2(\rho)$	E(r)	ρ	G(r)	V(r)
Bridging	211Nd-187B	0.10690	-0.00400	0.04015	0.03072	-0.03472
	212Nd-188B	0.10673	-0.00396	0.04009	0.03064	-0.03460
Chelating	211Nd-156B	0.02556	0.00069	0.01040	0.00570	-0.00501
	212Nd-147B	0.08599	-0.00128	0.03310	0.02278	-0.02406

Table S24. The average delocalization index (δ) for chelating and bridging M-B bonds.

Complexes	Bridging M-B	Chelating M-B
U	0.12	0.08
La	0.08	0.06
Ce	0.09	0.07
Pr	0.09	0.07
Nd	0.09	0.07

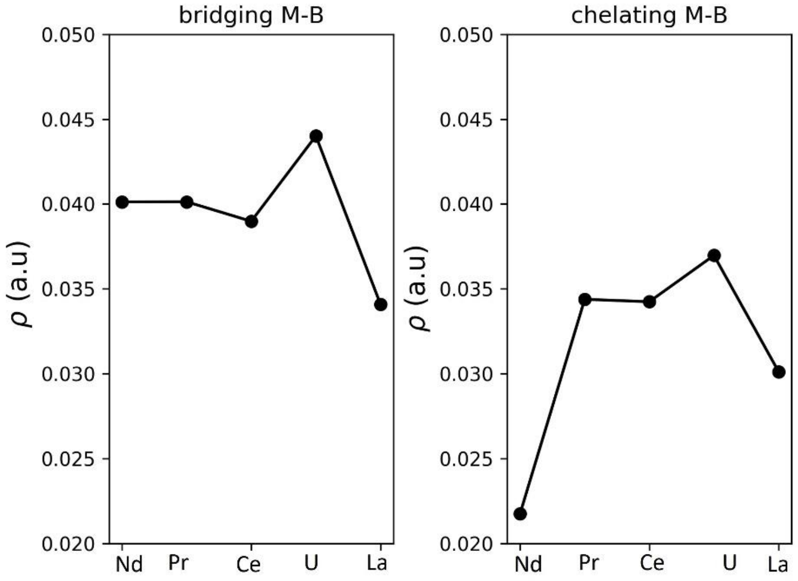

Figure S13. Average total electron density (ρ) at the bond critical point along the M-B bonds as a function of changing the metal.

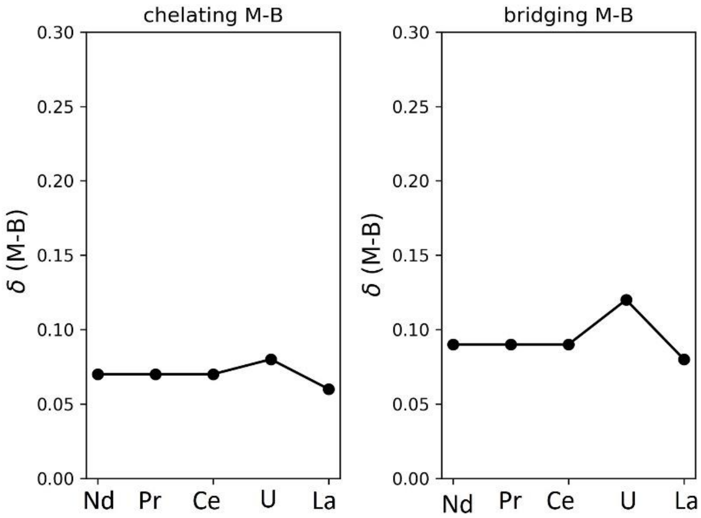

Figure S14. Average delocalization index (δ) as a function of changing the metal.

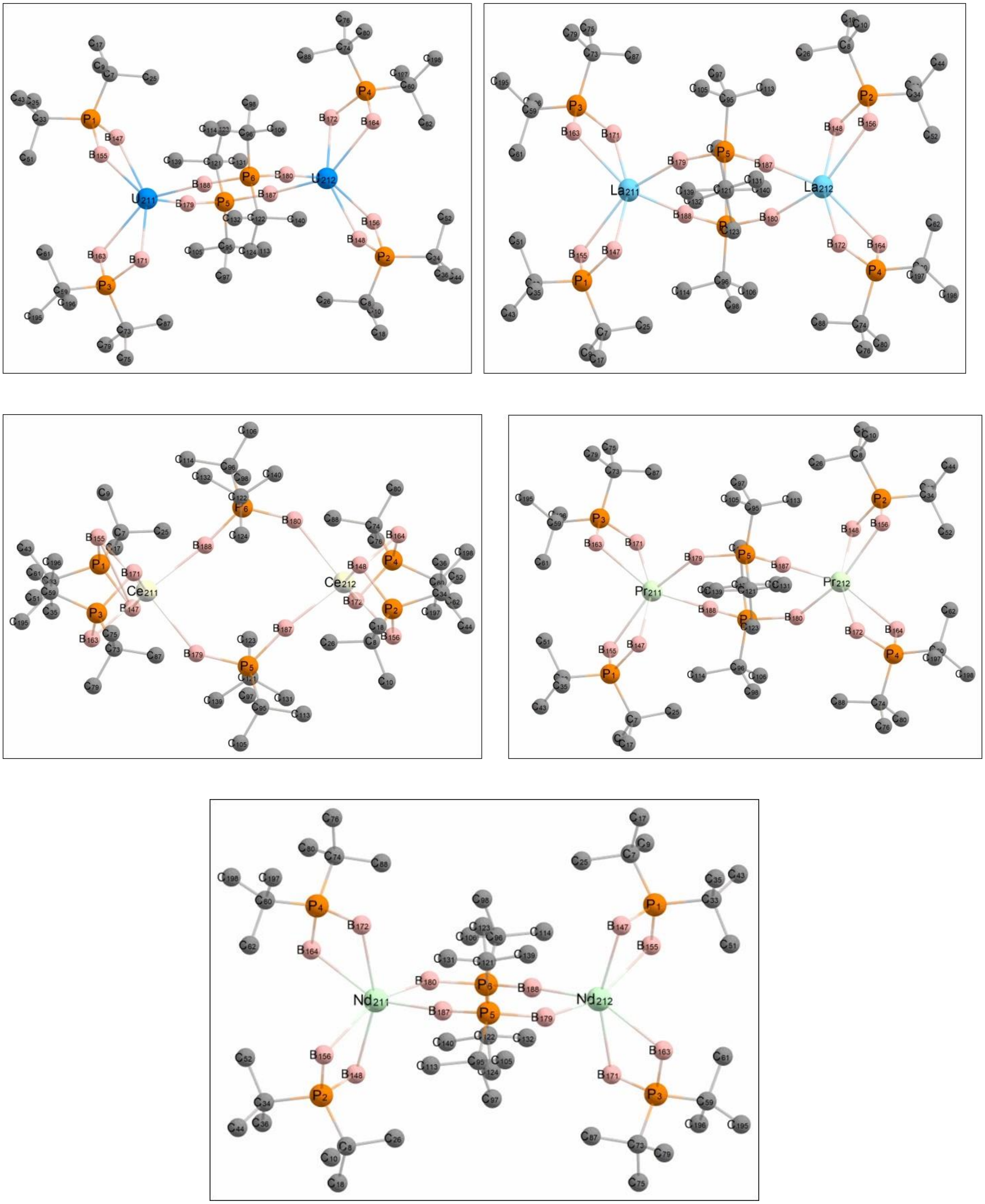

Figure S15. Optimized structures of $M_2(H_3BP^tBu_2BH_3)_6$ (M = U, La, Ce, Pr, and Nd). The hydrogen atoms were omitted for easier viewing of the atom labels.

5. NMR Spectra

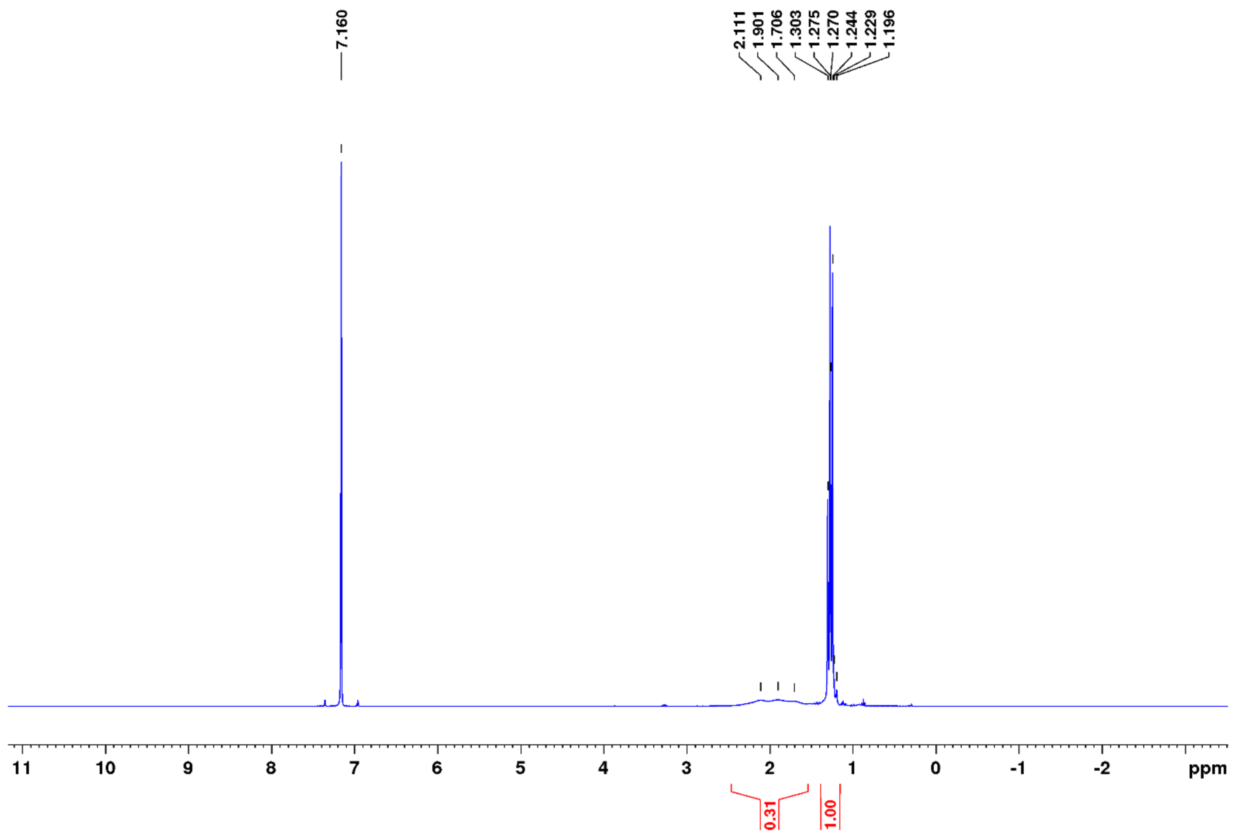

Figure S16. ^{1}H NMR spectrum of $La_2(H_3BP^tBu_2BH_3)_6$ in C_6D_6.

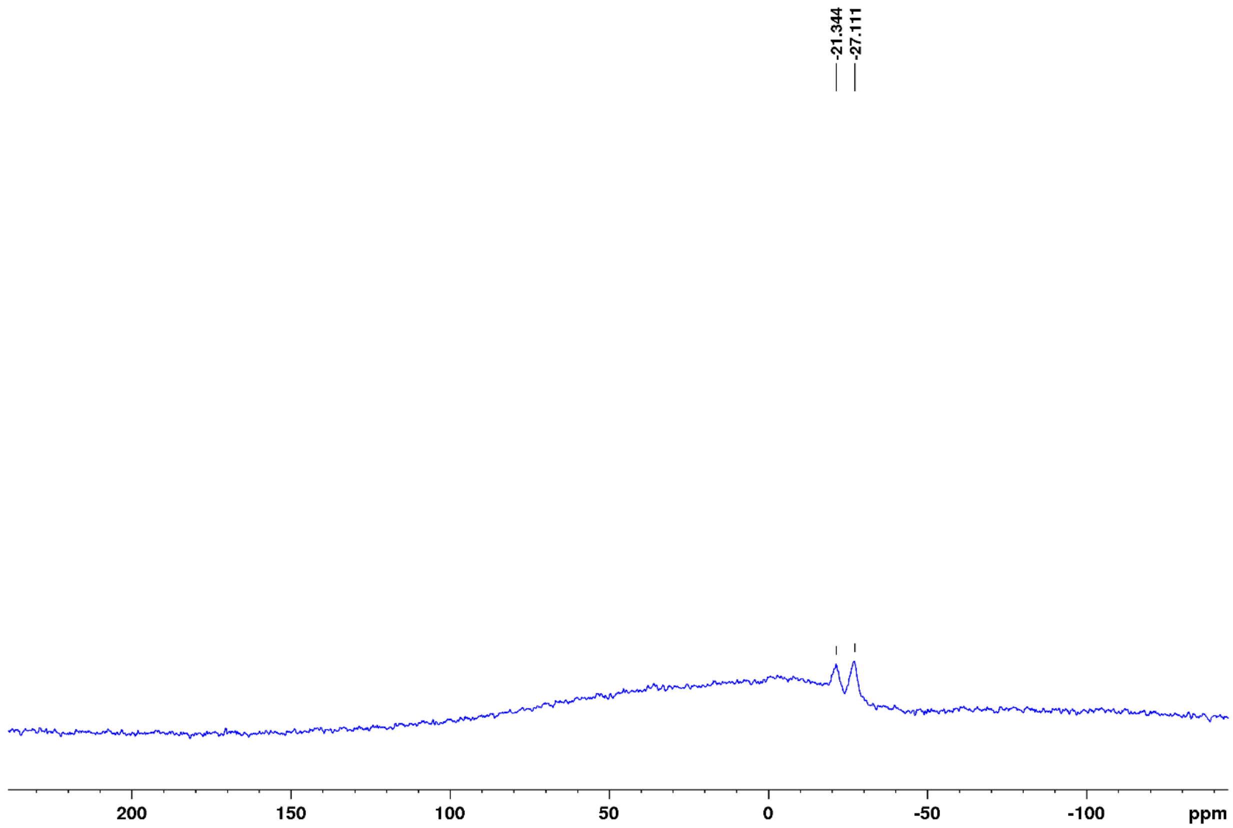

Figure S17. ^{11}B NMR spectrum of $La_2(H_3BP^tBu_2BH_3)_6$ in C_6D_6. The broad feature around δ 0 ppm is assigned to borosilicate inside of the instrument.

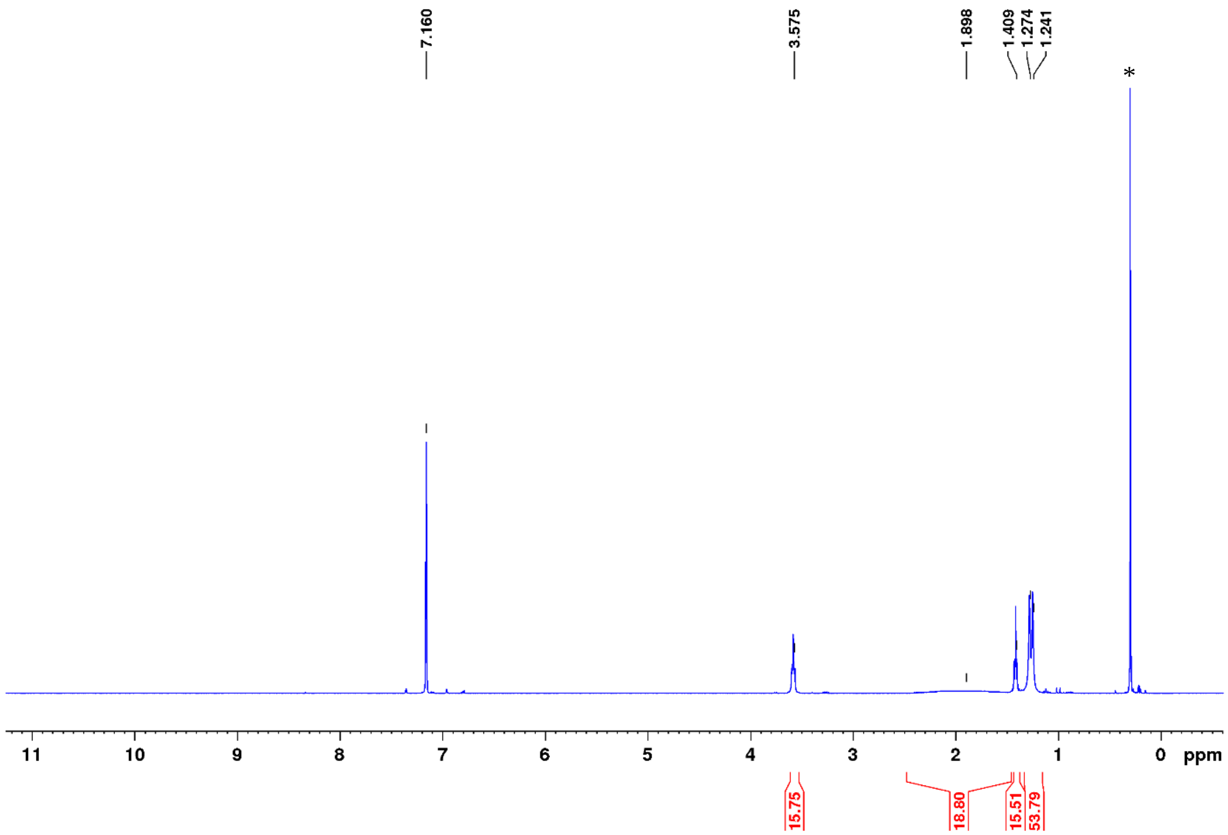

Figure S18. ^{1}H NMR spectrum of $La(H_3BP^tBu_2BH_3)_3(thf)_3$ in C_6D_6. The * symbol indicates a resonance assigned to silicone grease.

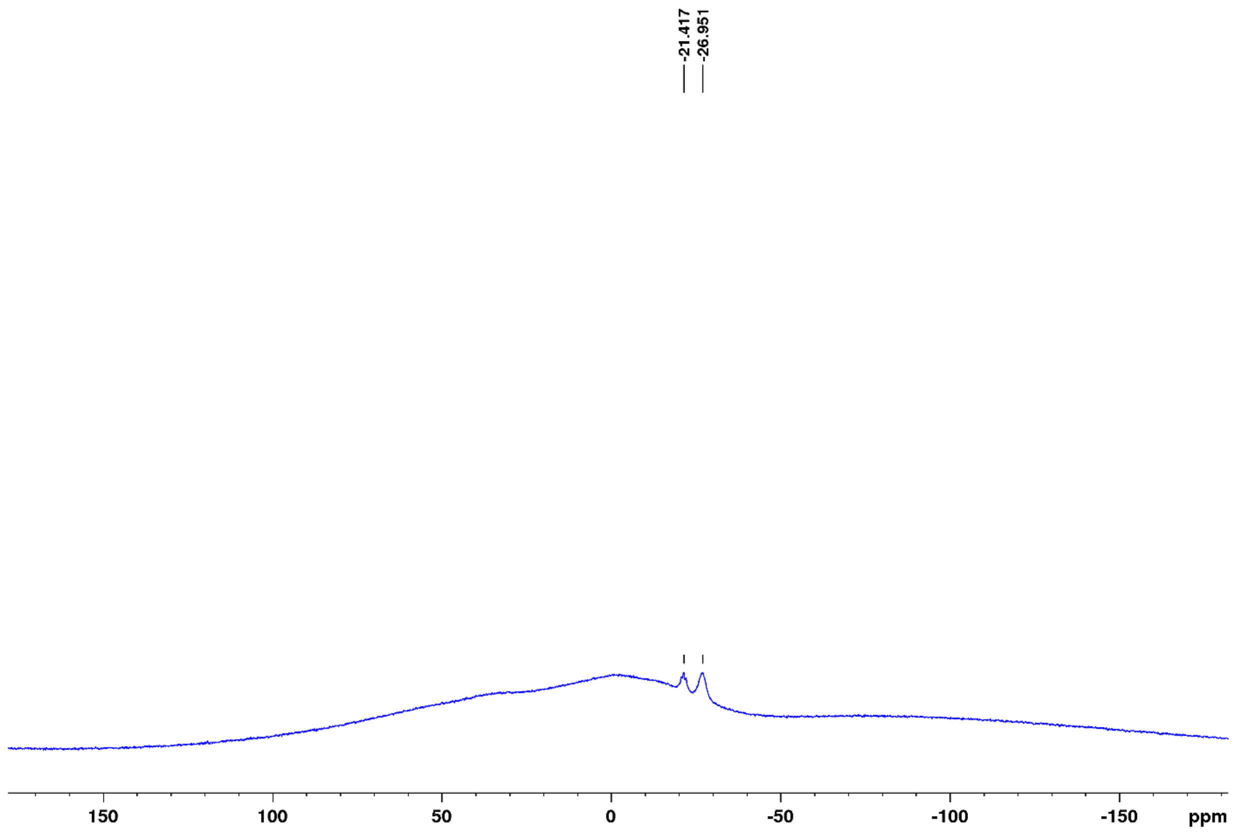

Figure S19. ^{11}B NMR spectrum of BH_3 resonances for $La(H_3BP^tBu_2BH_3)_3(thf)_3$ in C_6D_6. The broad feature around δ 0 ppm is assigned to borosilicate inside of the instrument.

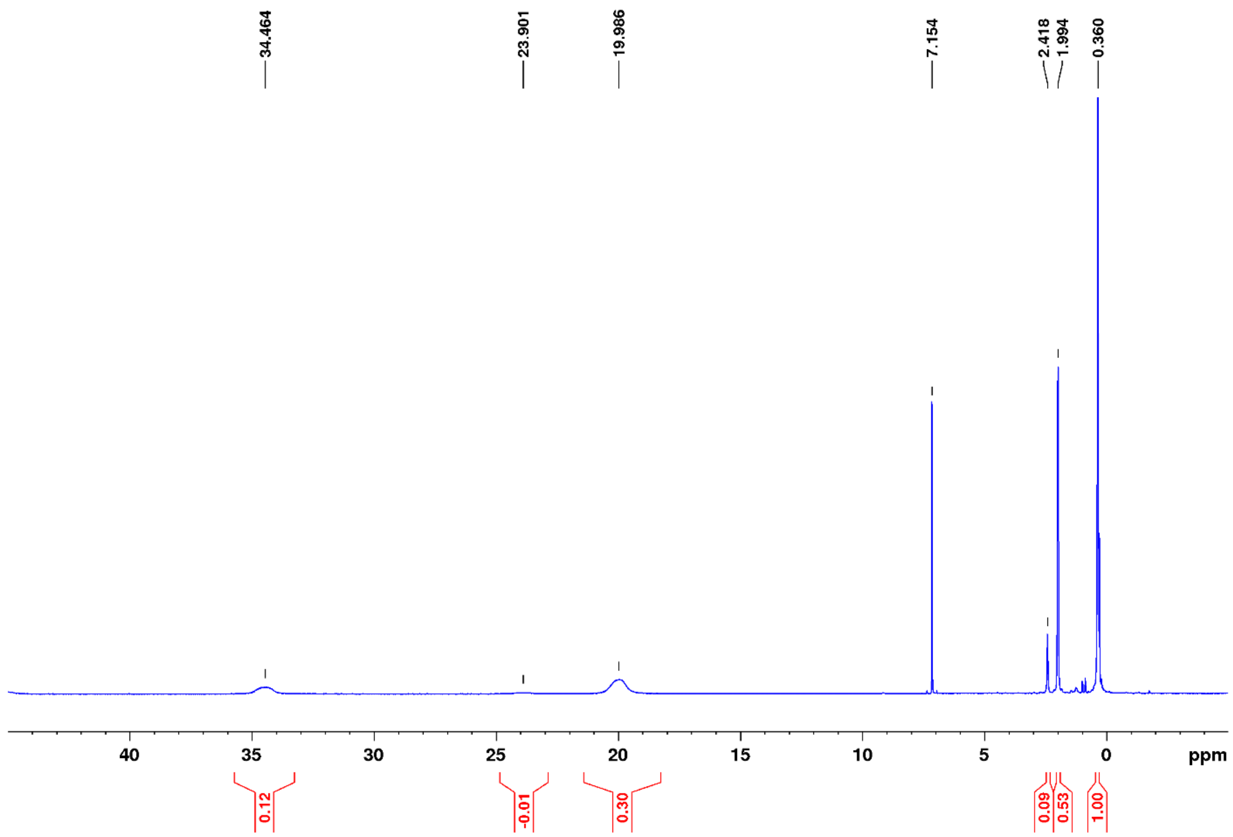

Figure S20. ^{1}H NMR spectrum of $Ce_2(H_3BP^tBu_2BH_3)_6$ in C_6D_6.

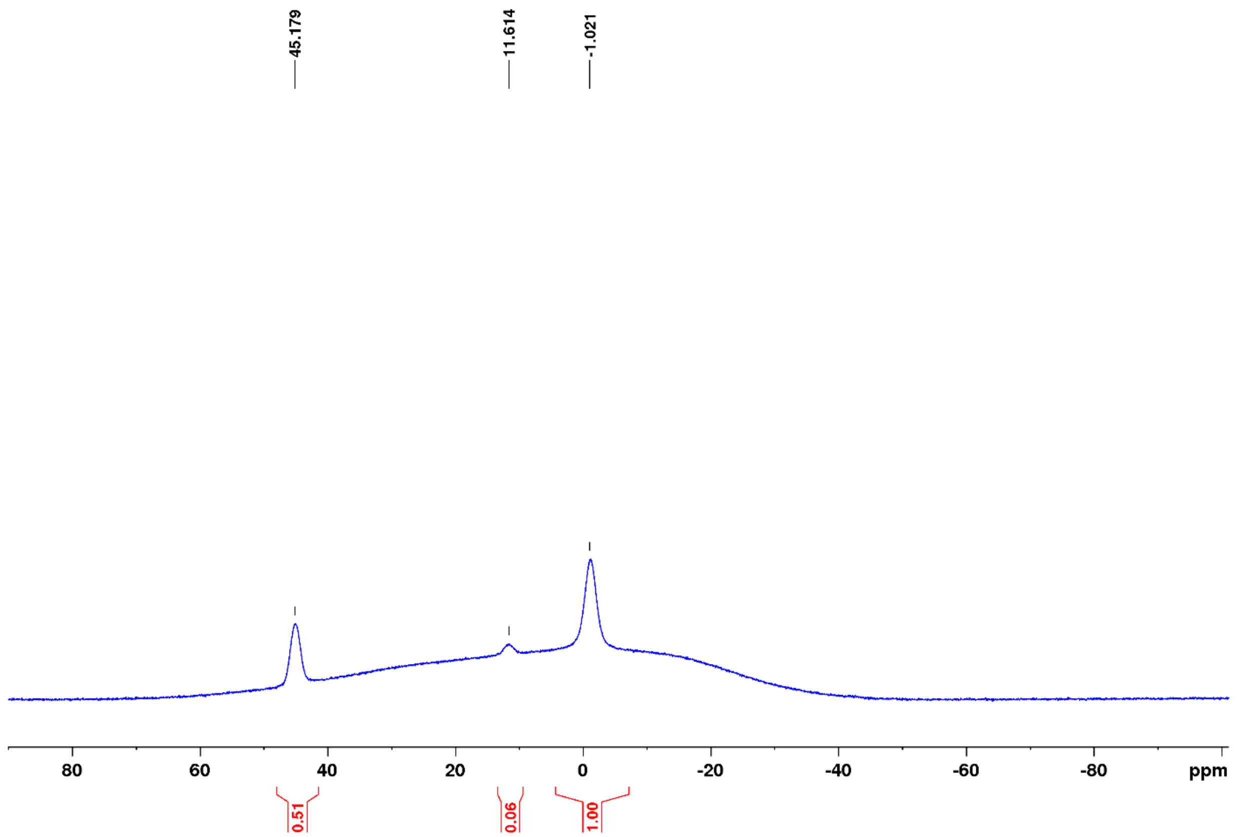

Figure S21. ^{11}B NMR spectrum of BH_3 resonances for $Ce_2(H_3BP^tBu_2BH_3)_6$ in C_6D_6. The broad feature around δ 0 ppm is assigned to borosilicate inside of the instrument.

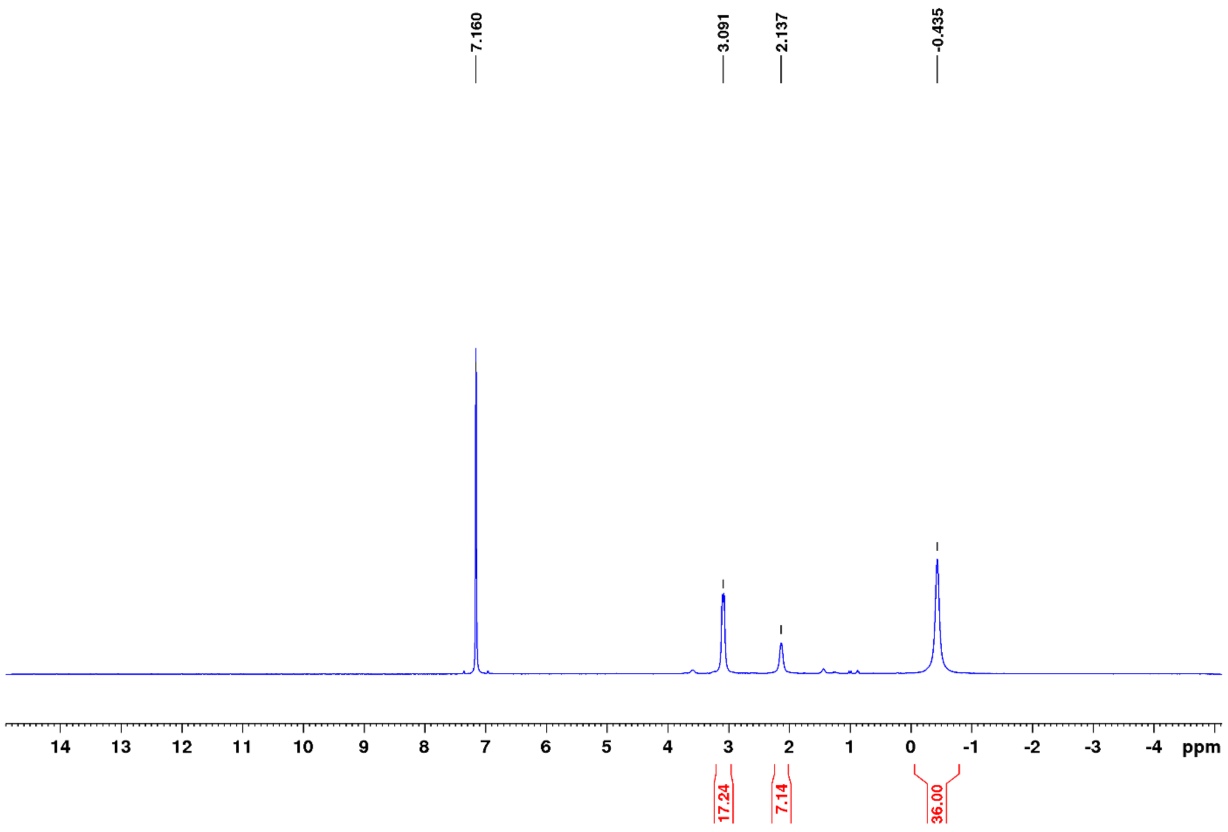

Figure S22. ^{1}H NMR spectrum of alkyl resonances for $Pr_2(H_3BP^{t}Bu_2BH_3)_6$ in C_6D_6.

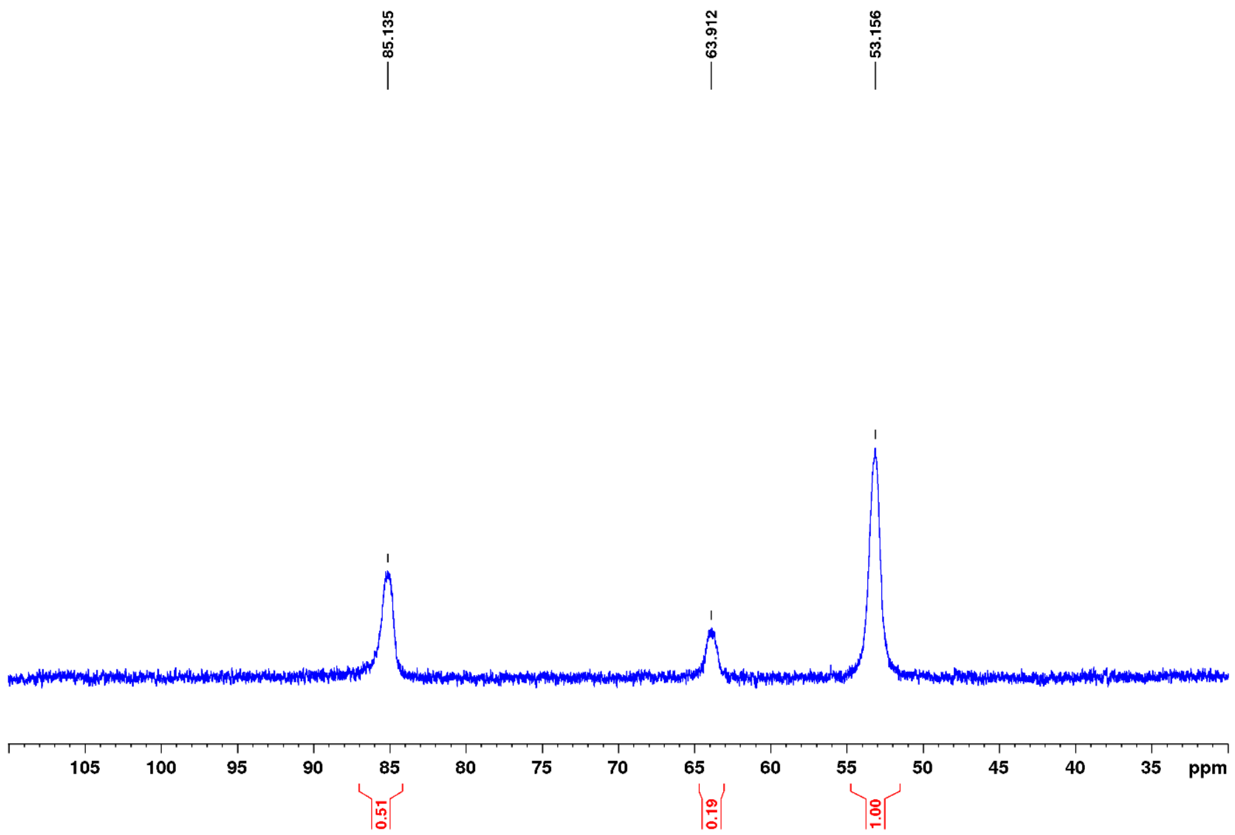

Figure S23. ^{1}H NMR spectrum (BH_3 resonances) of $Pr_2(H_3BP^tBu_2BH_3)_6$ in C_6D_6.

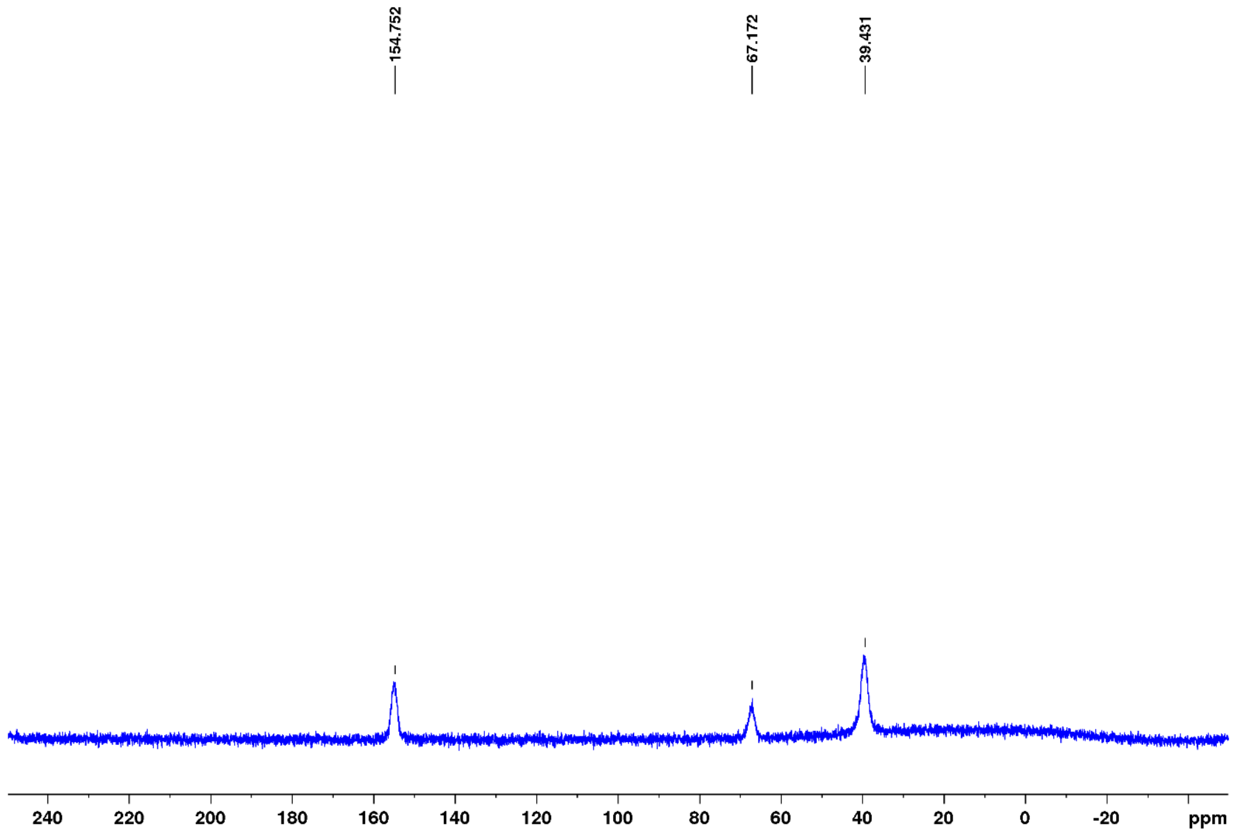

Figure S24. ^{11}B NMR spectrum of $Pr_2(H_3BP^tBu_2BH_3)_6$ in C_6D_6. The broad feature around δ 0 is assigned to borosilicate inside of the instrument.

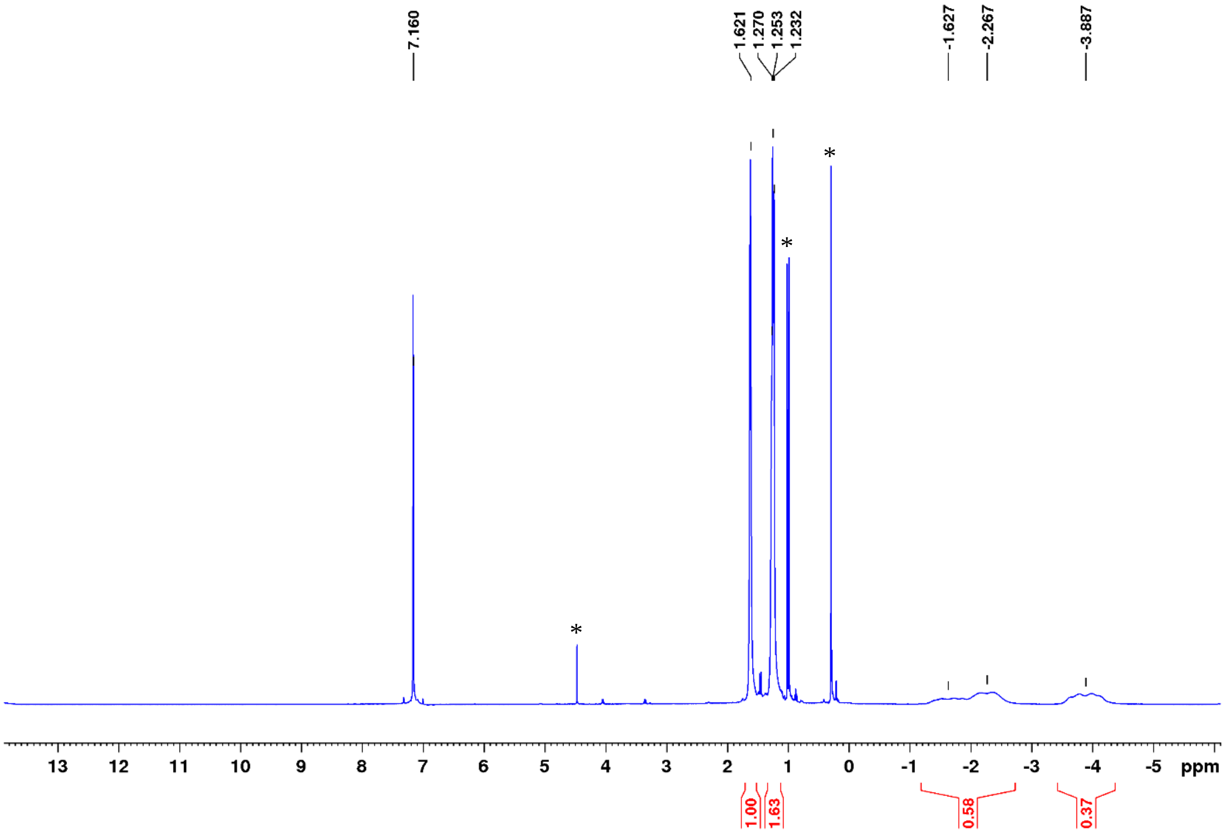

Figure S25. ^{1}H NMR spectrum of $Sm_2(H_3BP^tBu_2BH_3)_6$. The * symbol indicates resonances assigned to silicone grease and small hydrolysis impurities, as evident by the presence of H_2 at δ 4.47 ppm.

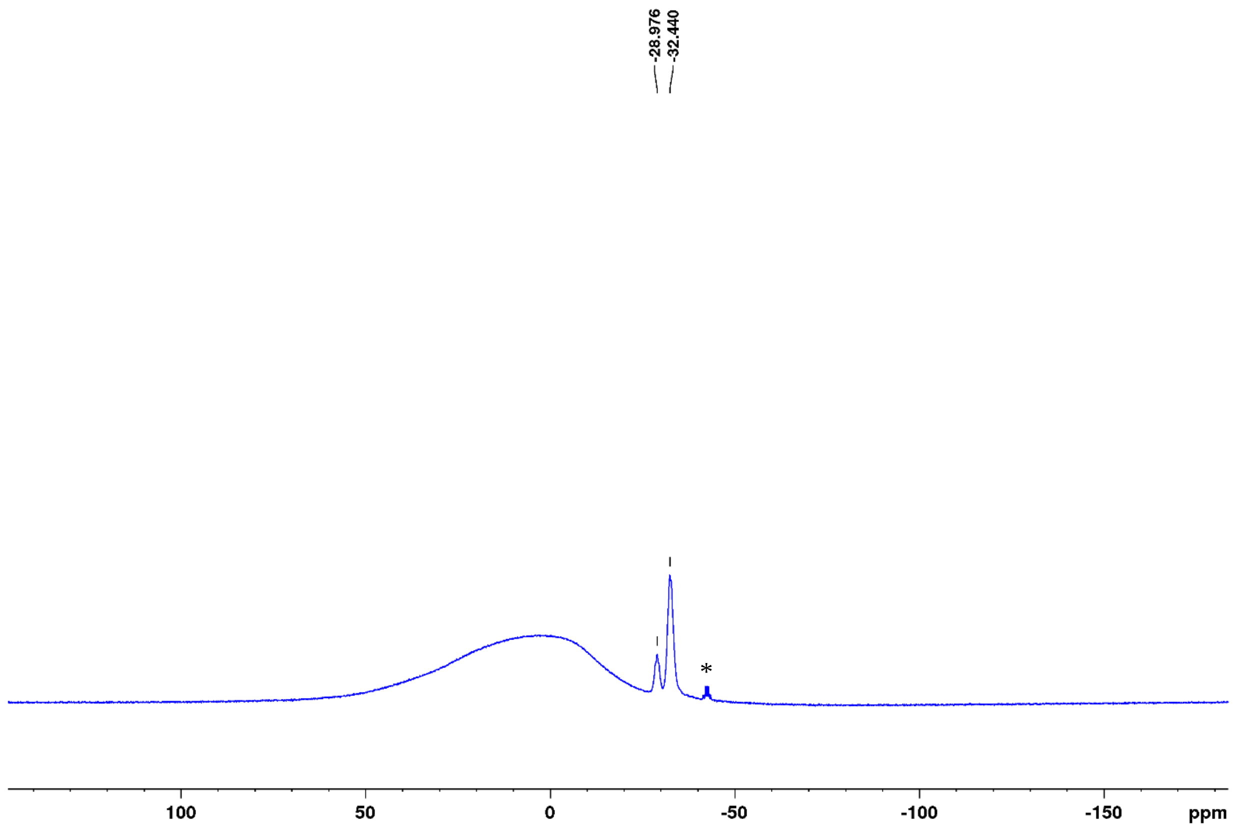

Figure S26. ^{11}B NMR spectrum of $Sm_2(H_3BP^tBu_2BH_3)_6$ in C_6D_6. The broad feature is assigned to borosilicate inside of the instrument. The * symbol indicates a resonance assigned to a small hydrolysis impurity.

6. IR Spectra

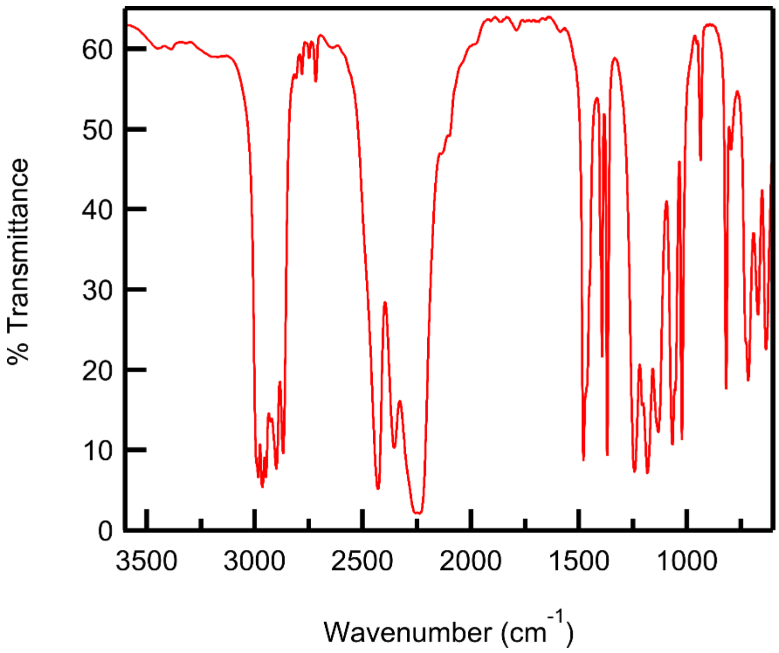

Figure 27. IR spectrum (KBr) of $La_2(H_3BP^tBu_2BH_3)_6$.

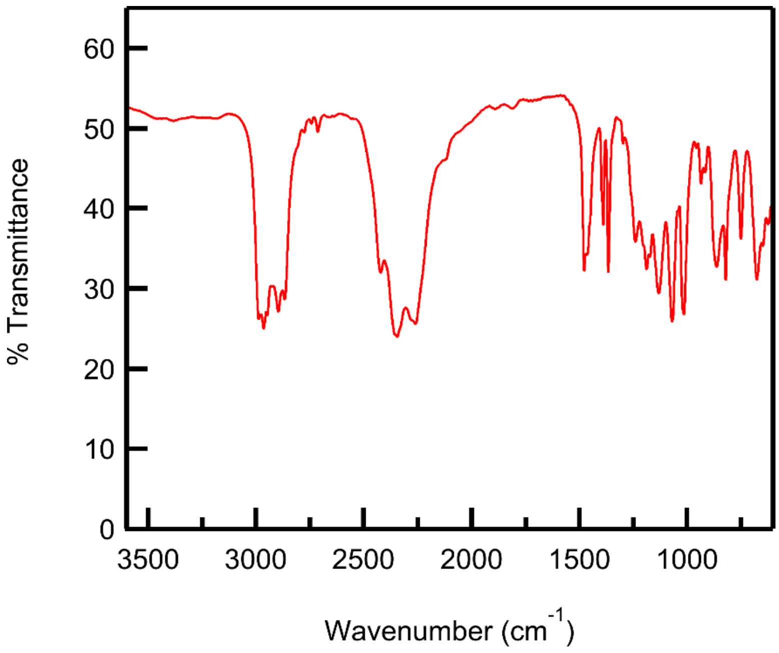

Figure S28. IR spectrum (KBr) of $La(H_3BP^tBu_2BH_3)_3(thf)_3$.

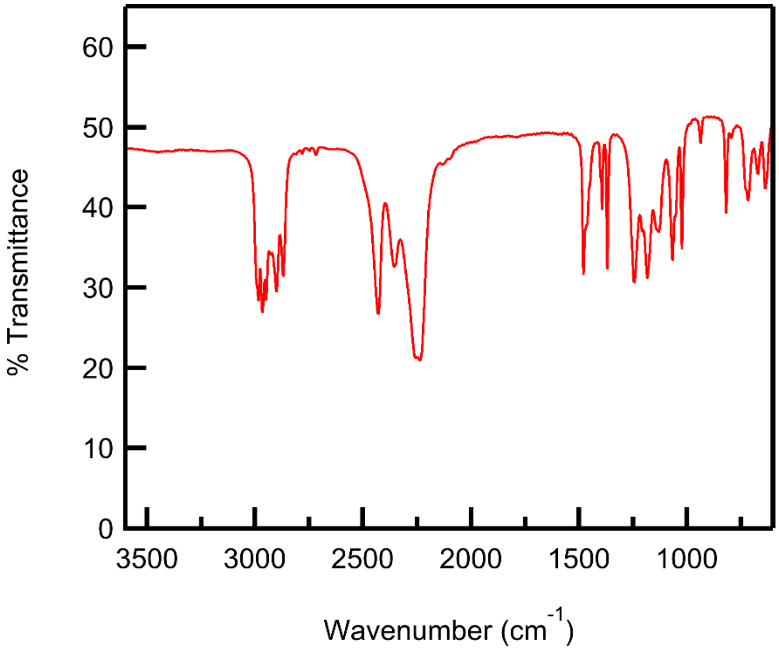

Figure S29. IR spectrum (KBr) of $Ce_2(H_3BP^tBu_2BH_3)_6$.

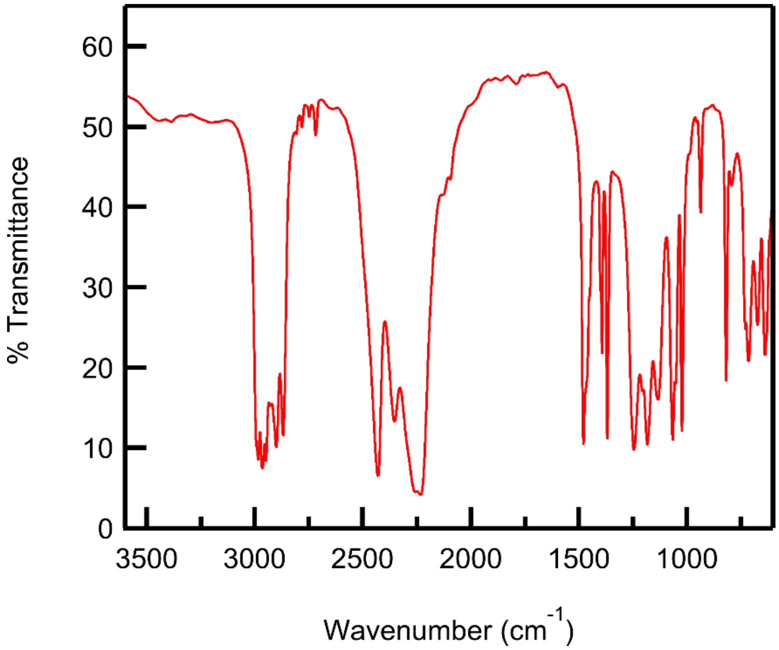

Figure S30. IR spectrum (KBr) of $Pr_2(H_3BP^tBu_2BH_3)_6$.

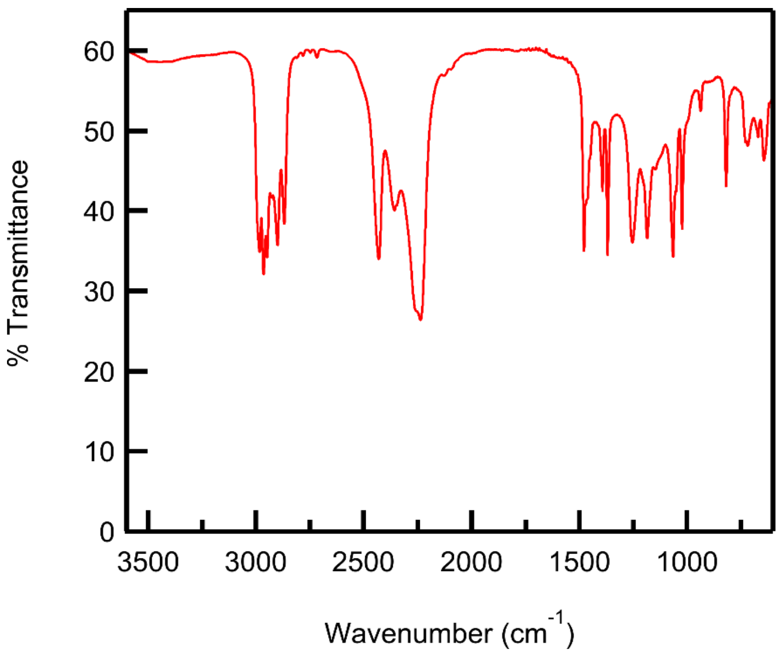

Figure S31. IR spectrum (KBr) of $Sm_2(H_3BP^tBu_2BH_3)_6$.

7. Supporting Information References

(1) Blake, A. V.; Fetrow, T. V.; Theiler, Z. J.; Vlaisavljevich, B.; Daly, S. R. *Chem. Comm.* **2018**, *54*, 5602-5605.

(2) Fetrow, T. V.; Bhowmick, R.; Achazi, A. J.; Blake, A. V.; Eckstrom, F. D.; Vlaisavljevich, B.; Daly, S. R. *Inorg. Chem.* **2020**, *59*, 48-61.

(3) Pauli, G. F.; Chen, S.-N.; Simmler, C.; Lankin, D. C.; Gödecke, T.; Jaki, B. U.; Friesen, J. B.; McAlpine, J. B.; Napolitano, J. G. *J. Med. Chem.* **2014**, *57*, 9220-9231.

(4) Van Geet, A. L. *Anal. Chem.* **1968**, *40*, 2227-2229.

(5) Tellinghuisen, J. *Biophys. Chem.* **2006**, *120*, 114-120.

(6) Tellinghuisen, J. *J. Chem. Ed.* **2015**, *92*, 864-870.

(7) Chen, P.; Dougan, B. A.; Zhang, X.; Wu, Y.-D.; Xue, Z.-L. *Polyhedron* **2013**, *58*, 30-38.

(8) Chen, T.; Wu, Z.; Li, L.; Sorasaenee, K. R.; Diminnie, J. B.; Pan, H.; Guzei, I. A.; Rheingold, A. L.; Xue, Z. *J. Am. Chem. Soc.* **1998**, *120*, 13519-13520.

(9) Chen, T.; Zhang, X.-H.; Wang, C.; Chen, S.; Wu, Z.; Li, L.; Sorasaenee, K. R.; Diminnie, J. B.; Pan, H.; Guzei, I. A.; Rheingold, A. L.; Wu, Y.-D.; Xue, Z.-L. *Organometallics* **2005**, *24*, 1214-1224.

(10) Sheldrick, G. M. *Acta Crystallogr., Sect. A Found. Adv.* **2015**, *71*, 3-8.

(11) Sheldrick, G. M. *Acta Crystallogr., Sect. A Found. Crystallogr.* **2008**, *64*, 112-122.

(12) Sheldrick, G. M. *Acta Crystallogr., Sect. C Struct. Chem.* **2015**, *71*, 3-8.

(13) Dolomanov, O. V.; Bourhis, L. J.; Gildea, R. J.; Howard, J. A. K.; Puschmann, H. *J. Appl. Crystallogr.* **2009**, *42*, 339-341.

(14) Macrae, C. F.; Sovago, I.; Cottrell, S. J.; Galek, P. T. A.; McCabe, P.; Pidcock, E.; Platings, M.; Shields, G. P.; Stevens, J. S.; Towler, M.; Wood, P. A. *J. Appl. Crystallogr.* **2020**, *53*, 226-235.

(15) Perdew, J. P.; Wang, Y. *Phys Rev B Condens Matter* **1992**, *45*, 13244-13249.

(16) Tao, J.; Perdew, J. P.; Staroverov, V. N.; Scuseria, G. E. *Phys. Rev. Lett.* **2003**, *91*, 146401/146401-146401/146404.

(17) Grimme, S.; Antony, J.; Ehrlich, S.; Krieg, H. *J. Chem. Phys.* **2010**, *132*, 154104/154101-154104/154119.

(18) Eichkorn, K.; Treutler, O.; Oehm, H.; Haeser, M.; Ahlrichs, R. *Chem. Phys. Lett.* **1995**, *240*, 283-290.

(19) Eichkorn, K.; Treutler, O.; Oehm, H.; Haeser, M.; Ahlrichs, R. *Chem. Phys. Lett.* **1995**, *242*, 652-660.

(20) Pracht, P.; Bohle, F.; Grimme, S. *Phys. Chem. Chem. Phys.* **2020**, *22*, 7169-7192.

(21) Klamt, A.; Schueuermann, G. *J. Chem. Soc., Perkin Trans. 2* **1993**, 799-805.

(22) Eichkorn, K.; Weigend, F.; Treutler, O.; Ahlrichs, R. *Theor. Chem. Acc.* **1997**, *97*, 119-124.

(23) Kuechle, W.; Dolg, M.; Stoll, H.; Preuss, H. *J. Chem. Phys.* **1994**, *100*, 7535-7542.

(24) Cao, X.; Dolg, M.; Stoll, H. *J. Chem. Phys.* **2003**, *118*, 487-496.

(25) Dolg, M.; Stoll, H.; Preuss, H. *J. Chem. Phys.* **1989**, *90*, 1730-1734.

(26) Weigend, F.; Ahlrichs, R. *Phys. Chem. Chem. Phys.* **2005**, *7*, 3297-3305.

(27) Dolg, M.; Stoll, H.; Preuss, H. *Theor. Chim. Acta* **1993**, *85*, 441-450.

(28) Cao, X.; Dolg, M. *J. Chem. Phys.* **2001**, *115*, 7348-7355.

(29) Weigend, F.; Haser, M.; Patzelt, H.; Ahlrichs, R. *Chem. Phys. Lett.* **1998**, *294*, 143-152.

(30) Ribeiro, R. F.; Marenich, A. V.; Cramer, C. J.; Truhlar, D. G. *J. Phys. Chem. B* **2011**, *115*, 14556-14562.

(31) Bader, R. F. W. *Chem. Rev.* **1991**, *91*, 893-928.

(32) Lu, T.; Chen, F. *J. Comput. Chem.* **2012**, *33*, 580-592.

(33) Huang, P.-W.; Wang, C.-Z.; Wu, Q.-Y.; Lan, J.-H.; Song, G.; Chai, Z.-F.; Shi, W.-Q. *Phys. Chem. Chem. Phys.* **2018**, *20*, 14031-14039.

(34) Espinosa, E.; Alkorta, I.; Elguero, J.; Molins, E. *J. Chem. Phys.* **2002**, *117*, 5529-5542.

(35) Kerridge, A. *RSC Adv.* **2014**, *4*, 12078-12086.
(36) Kerridge, A. *Chem. Commun.* **2017**, *53*, 6685-6695.
(37) Kohler, L.; Patzschke, M.; Schmidt, M.; Stumpf, T.; Marz, J. *Chem. - Eur. J.* **2021**, *27*, 18058-18065.
